# Boosting Hydrogen Storage Performance of MgH_2_ by Oxygen Vacancy-Rich H-V_2_O_5_ Nanosheet as an Excited H-Pump

**DOI:** 10.1007/s40820-024-01375-8

**Published:** 2024-03-21

**Authors:** Li Ren, Yinghui Li, Zi Li, Xi Lin, Chong Lu, Wenjiang Ding, Jianxin Zou

**Affiliations:** 1 National Engineering Research Center of Light Alloys Net Forming & State Key Laboratory of Metal Matrix Composites, Shanghai Jiao Tong University, Shanghai, 200240 People’s Republic of China; 2 Shanghai Engineering Research Center of Mg Materials and Applications & School of Materials Science and Engineering, Shanghai Jiao Tong University, Shanghai, 200240 People’s Republic of China; 3 Center of Hydrogen Science, Shanghai Jiao Tong University, Shanghai, 200240 People’s Republic of China; 4 Instrumental Analysis Center of SJTU, Shanghai Jiao Tong University, Shanghai, 200240 People’s Republic of China

**Keywords:** Hydrogen storage, MgH_2_, V_2_O_5_ nanosheets, Oxygen vacancies, VH_2_

## Abstract

MgH_2_ is a promising high-capacity solid-state hydrogen storage material, while its application is greatly hindered by the high desorption temperature and sluggish kinetics. Herein, intertwined 2D oxygen vacancy-rich V_2_O_5_ nanosheets (H-V_2_O_5_) are specifically designed and used as catalysts to improve the hydrogen storage properties of MgH_2_. The as-prepared MgH_2_-H-V_2_O_5_ composites exhibit low desorption temperatures (*T*_onset_ = 185 °C) with a hydrogen capacity of 6.54 wt%, fast kinetics (*E*_a_ = 84.55 ± 1.37 kJ mol^−1^ H_2_ for desorption), and long cycling stability. Impressively, hydrogen absorption can be achieved at a temperature as low as 30 °C with a capacity of 2.38 wt% within 60 min. Moreover, the composites maintain a capacity retention rate of ~ 99% after 100 cycles at 275 °C. Experimental studies and theoretical calculations demonstrate that the in-situ formed VH_2_/V catalysts, unique 2D structure of H-V_2_O_5_ nanosheets, and abundant oxygen vacancies positively contribute to the improved hydrogen sorption properties. Notably, the existence of oxygen vacancies plays a double role, which could not only directly accelerate the hydrogen ab/de-sorption rate of MgH_2_, but also indirectly affect the activity of the catalytic phase VH_2_/V, thereby further boosting the hydrogen storage performance of MgH_2_. This work highlights an oxygen vacancy excited “hydrogen pump” effect of VH_2_/V on the hydrogen sorption of Mg/MgH_2_. The strategy developed here may pave a new way toward the development of oxygen vacancy-rich transition metal oxides catalyzed hydride systems.

**Supplementary Information:**

The online version contains supplementary material available at 10.1007/s40820-024-01375-8.

## Highlights

- Graphene-like 2D V_2_O_5_ nanosheets rich in oxygen vacancies are designed as multi-functional catalysts to fabricate MgH_2_-H-V_2_O_5_ composites.
- Hydrogen release starts from 185 °C and capacity retention is as high as 99% after 100 cycles at 275 °C.
- The composites present rapid kinetics and impressive hydrogen absorption capability at near room temperature.
- The oxygen vacancies could directly enhance kinetics of MgH_2_ while indirectly exciting “hydrogen pump” effect of VH_2_/V.

**Supplementary Information:**

The online version contains supplementary material available at 10.1007/s40820-024-01375-8.

## Graphical abstract

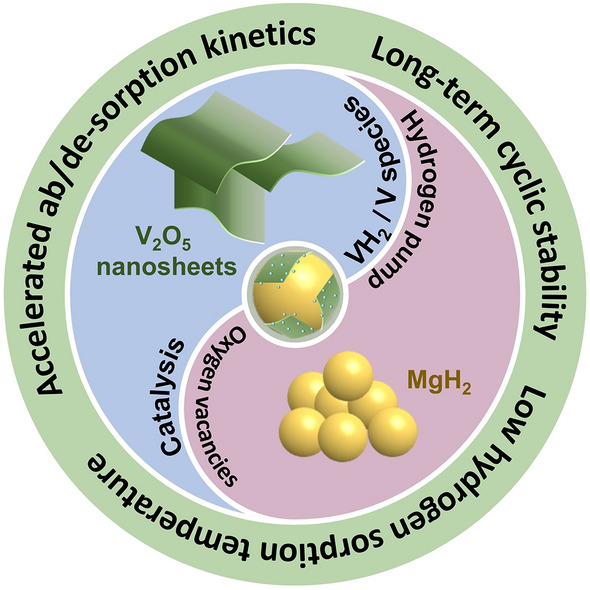

## Introduction

With the depletion of fossil fuels and global warming, there is an urgent need to seek green, clean, and high-efficiency energy resources [1–4]. Hydrogen is considered as a potential candidate to replace fossil fuels, due to its high gravimetric energy density (142 MJ kg^−1^) and environmental-friendly nature [5, 6]. Nevertheless, safe and efficient hydrogen storage technology is now the bottleneck constraining the development of the hydrogen economy [7]. Compared with compressed gaseous and cryogenic liquid hydrogen storage technologies, solid-state hydrogen storage is deemed to be a safe and efficient method [8]. Magnesium hydride (MgH_2_), as one of the most promising solid-state hydrogen storage materials, has received extensive attention, due to the elemental abundance of Mg on earth, high storage capacity (7.6 wt%, 110 kg H_2_ m^−3^), good reversibility, and non-toxicity [9–11]. According to the targets set by DOE, the reactivity and safety of candidate hydrogen storage materials are key parameters for their mobile and stationary applications. MgH_2_ is one of the potential solid-state hydrogen storage media that has been evaluated for use in a series of applications including hydrogen-fueled vehicles, forklift trucks, baggage and pushback tractors, stationary power, portable power, and grid energy storage applications to promote efficient power generation of the intermittent renewable energy sources such as wind and solar. Additionally, MgH_2_ could provide H_2_ for fuel cell (FC) to generate electricity through hydrolysis. The development of personal vehicle equipped with MgH_2_-reactor and FC is expected to alleviate energy crisis and environmental pollution. Recently, a tonnage Mg-based solid-state hydrogen storage trailer was developed, marking a new breakthrough in the field of hydrogen storage and transportation [12]. However, the sluggish kinetics and stable thermodynamics result in the high operating temperature of over 300 °C for MgH_2_ [13], hindering its large-scale commercial application for on-board or stationary hydrogen storage.

In the past decades, intensive endeavors have been made towards the improvement of the hydrogen storage performance of MgH_2_, such as nano-structuring [14, 15], alloying [16–19], and catalyzing [20–22]. The introduction of transition metal-based catalysts, which have a unique 3d electronic structure, is deemed to be the most effective method to improve the kinetics of MgH_2_. Benefitting from multi-valance and high catalytic activity, vanadium (V) metal and its corresponding oxide counterparts are often used as catalysts for MgH_2_ to improve its performance. However, limited by the high ductility and relatively low activity of metal V, V-based oxides offer a wider range of applications than pure metals for the low cost and abundant resource. Furthermore, due to the high hardness, V-based oxides could be used as grinding aids during the process of ball-milling, which may promote a uniform distribution of the catalyst and a finer microstructure of MgH_2_/Mg [23]. Among numerous vanadium-based oxides, V_2_O_5_, presenting a layered structure, is a promising catalyst for enhancing the hydrogen storage performance of MgH_2_/Mg. However, the insufficient contact between the bulk V_2_O_5_ and MgH_2_ resulted in its limited catalytic capability.

Some strategies have been implemented to further improve the catalytic activity of V-based catalysts and a viable approach is to fabricate nanostructure to increase the contact area with MgH_2_/Mg [24–26]. Compared to the bulk counterparts [25, 27–29], nanostructured V_2_O_5_ such as nanosheets could offer favorable properties, including shortened hydrogen diffusion distance, enlarged interfacial contact area with MgH_2_/Mg, and abundant exposed active sites. Additionally, it is worth emphasizing that heavily oxidized V cannot provide enough electrons to compensate for Mg-H breakage when H diffuses from Mg to V, which could not fully utilize the catalytic activity of V_2_O_5_. Moreover, the possible reaction between MgH_2_ and V_2_O_5_ would result in the consumption of Mg and continuous degradation of reversible hydrogen storage capacity, which may be ameliorated via the introduction of oxygen vacancies. The computational investigation further demonstrated that partially oxidized transition metal is helpful not only in facilitating hydrogen diffusion but also in reducing the H–H coupling barrier [30–33]. In the case of oxygen vacancy-rich 2D V_2_O_5_ nanosheets, the presence of oxygen vacancies could lead to a shift in the position of the Fermi energy level towards the valence band, resulting in an increase in the number of available states for electron transfer. Numerous experimental and theoretical studies have revealed that oxygen vacancies can boost hydrogen diffusion and decrease the activation energy of hydrogen sorption reactions, because oxygen vacancies can accelerate the electron transfer [31–35]. However, to the best of our knowledge, given the numerous researches on Mg-based hydrogen storage composites, it still lacks studies regarding the influence of oxygen vacancies-rich V_2_O_5_ nanosheets on the hydrogen storage performances of MgH_2_ and the specific mechanism of the oxygen vacancy remains vague.

Herein, ultrathin hydrogenated V_2_O_5_ nanosheets with abundant oxygen vacancies were prepared by the solvothermal method and subsequent hydrogenation strategy, which were then used as catalysts to improve the hydrogen storage properties of MgH_2_. The resulting MgH_2_-H-V_2_O_5_ composites exhibited superior hydrogen storage performances with reduced desorption temperatures (T_onset_ = 185 °C), rapid kinetics (*E*_*a*_ = 84.55 kJ mol^−1^ H_2_ for desorption), and long-term cyclic stability (capacity retention up to 99% after 100 cycles). Particularly, MgH_2_-H-V_2_O_5_ composites exhibit excellent hydrogen absorption capability at room temperature, which could absorb 2.38 wt% within 60 min at 30 °C. Experimental analysis and theoretical calculations indicated that the unique 2D structure of H-V_2_O_5_ nanosheets with abundant oxygen vacancies and in-situ formed V/VH_2_ species are responsible for the improved hydrogen storage performances of MgH_2_. The nanosheet-like H-V_2_O_5_ can expose more active sites and hydrogen/electron diffusion pathways due to the highly exposed surfaces of the unique anisotropic layer structure formed during the solvothermal process, thereby promoting hydrogen storage properties. Furthermore, part of H-V_2_O_5_ will be reduced to metallic vanadium, which could participate in the de/re-hydrogenation process of MgH_2_ by the phase transition between VH_2_ and V. More importantly, the presence of oxygen vacancies could accelerate electron transfer and inspire the “hydrogen pump” effect of VH_2_/V, promoting the dehydrogenation of VH_2_ and MgH_2_ and reducing the energy barrier for hydrogen dissociation and recombination. This work paves a new way for improving the cycling stability and kinetics of MgH_2_ through the defect engineering strategy by introducing oxygen vacancies in the catalysts.

## Experiment Section

### Sample Preparation

#### ***Synthesis of Hydrogenated V***_***2***_***O***_***5***_*** (H-V***_***2***_***O***_***5***_***)***

The hydrogenated V_2_O_5_ nanosheets were prepared by a hydrothermal reaction and subsequent heat treatment in hydrogen. In a typical procedure, 0.234 g of NH_4_VO_3_ powders (Sigma-Aldrich) were dissolved into 39 mL of distilled water under stirring. Then, 1 mL of concentrated HCl (≥ 37%) was added to the solution dropwise forming a homogeneous solution (the titration rate of HCl is about 6 ~ 8 mL min^−1^), which was then sealed into a 50 mL Teflon-lined autoclave. The V_2_O_5_·xH_2_O gel was obtained after a hydrothermal reaction at 200 °C for 1 h, which was freeze-dried for 72 h. To eliminate the effects of absorbed water on the hydrogen storage performance of MgH_2_, the V_2_O_5_·xH_2_O was annealed in air at 350 °C to generate V_2_O_5_-350air powder. Finally, the V_2_O_5_-350air powder was hydrogenated in hydrogen at different temperatures for 2 h to obtain H-V_2_O_5_ nanosheets. The temperature was set as 200 and 300 °C (samples denoted as H-V_2_O_5_-200 and H-V_2_O_5_-300, respectively).

#### ***Synthesis of MgH***_***2***_***-H-V***_***2***_***O***_***5***_*** Composites***

The H-V_2_O_5_ nanosheets were mixed with commercial MgH_2_ (Shanghai Mg Powder Technology Limited) to synthesize Mg-based hydrogen storage composites (named as MgH_2_-H-V_2_O_5_, weight ration = 90:10) in an argon glove box to inhibit the oxidation of samples. Afterward, the composites were milled for 12 h in a high-energy ball mill (ball-to-powder ratio = 80:1) at 400 rpm under the protection of 1.5 MPa hydrogen. For comparison, the pristine MgH_2_-BM, MgH_2_-10 wt% V_2_O_5_·xH_2_O, and MgH_2_-10 wt% V_2_O_5_-350air were also obtained by milling pristine MgH_2_, the mixture of MgH_2_ and V_2_O_5_·xH_2_O (V_2_O_5_-350air) under the same conditions.

### Characterization and Measurements

X-ray diffraction (XRD, Mini Flex 600) was performed with Cu-Kα radiation (40 kV, 15 mA) at a scan rate of 5° min^−1^ in the range of 5-80°. The samples were prepared in an Ar atmosphere glovebox and sealed in a custom-designed holder covered by Scotch tape to avoid possible oxidation during the XRD tests. The structure and morphology were studied by transmission electron microscopy (TEM, FEI Talos F200X G2) and scanning electron microscopy (SEM, MIRA3 LHM) equipped with an energy-dispersive X-ray spectrometer (EDS). For TEM observations, the preparation of samples was in the glovebox. The samples were dispersed in cyclohexane, sonicated, dropped cast on a copper grid and rapidly transferred to the equipment. The preparation conditions of samples for SEM analyses were similar to the TEM observations. The only difference was that the samples were dispersed in cyclohexane and dropped to the silicon wafer. X-ray photoelectron spectroscopy (XPS, Kratos AXIS Ultra DLD) was conducted to analyze the valence state and chemical bonding nature of constituent elements of MgH_2_-H-V_2_O_5_ composites. For XPS analyses, a special air-proof transfer vessel was used to transfer the samples from glovebox to the equipment. Thermal gravity analysis (TGA) (PerkinElmer DSC 8000) was performed in air with a heating rate of 10 °C min^−1^ from room temperature to 700 °C to quantify the content of oxygen vacancies and the entire testing process takes 67.5 min. Raman spectra of samples were recorded with a confocal Raman microscope (Renishaw inVia Qontor, UK). The Brunner–Emmet–Teller (BET) surface area of samples was measured with N_2_ at 77 K using an Autosorb-IQ3 apparatus.

Hydrogen sorption behaviors of composites were carried out using a commercialized sievert’s apparatus (HPSA-auto device, China). The temperature-programmed desorption (TPD) curves were obtained for studying hydrogen evolution under vacuum at a heating rate of 2 °C min^−1^ up to 350 °C. The isothermal hydrogenation with an initial H_2_ pressure of 3 MPa and isothermal dehydrogenation under 0.01 MPa initial hydrogen back-pressure were carried out by rapidly heating the sample to preset temperature and stabilizing at this temperature during the entire test. As for cycling test, the desorption behavior was performed under 0.01 MPa initial hydrogen back-pressure at 275 °C in 10 min, and absorption behavior was performed under 3 MPa initial H_2_ pressure at 275 °C in 10 min. The thermodynamic properties of the composites are determined by the pressure-composition-isothermal curve (PCI). To analyze the desorption kinetic properties, the composites were heated from room temperature to 500 °C with the heating rates of 3, 5, 7, and 10 °C min^−1^ under a flowing Ar atmosphere using differential scanning calorimetry (DSC, NETZSCH STA 449 F3) measurements (at different heating rates, the test duration is 158.3 min (3 °C min^−1^), 95 min (5 °C min^−1^), 67.8 min (7 °C min^−1^), and 47.5 min (10 °C min^−1^), respectively), and the obtained peak temperatures were fitted using the Kissinger equation to calculate the apparent activation energy of the dehydrogenation reaction.

### Theoretical Calculations

Density functional theory (DFT) calculations were carried out in Vienna ab initio simulation package (VASP) [36, 37]. Perdew-Burke-Ernzerhof (PBE) functionals and generalized gradient approximation (GGA) form were used to describe the exchange–correlation energy [38]. The projected augmented wave (PAW) potentials [39, 40] were chosen to describe the ionic cores, and valence electrons were taken into account using a plane wave basis set with a kinetic energy cutoff of 520 eV. The GGA + U method was adopted in our calculations. The value of the effective Hubbard U was set as 4.72 for V. Partial occupancies of the Kohn–Sham orbitals were allowed using the Gaussian smearing method with a width of 0.05 eV. The structures were relaxed until the forces and total energy on all atoms converged to less than 0.05 eV Å^−1^ and 1 × 10^–5^ eV. The Brillouin zone integration is performed using 2 × 2 × 1 Monkhorst–Pack k-point sampling for a structure.

## Results and Discussion

### Characterization of the As-Synthesized H-V_2_O_5_ Nanosheets

**Fig. 1:**
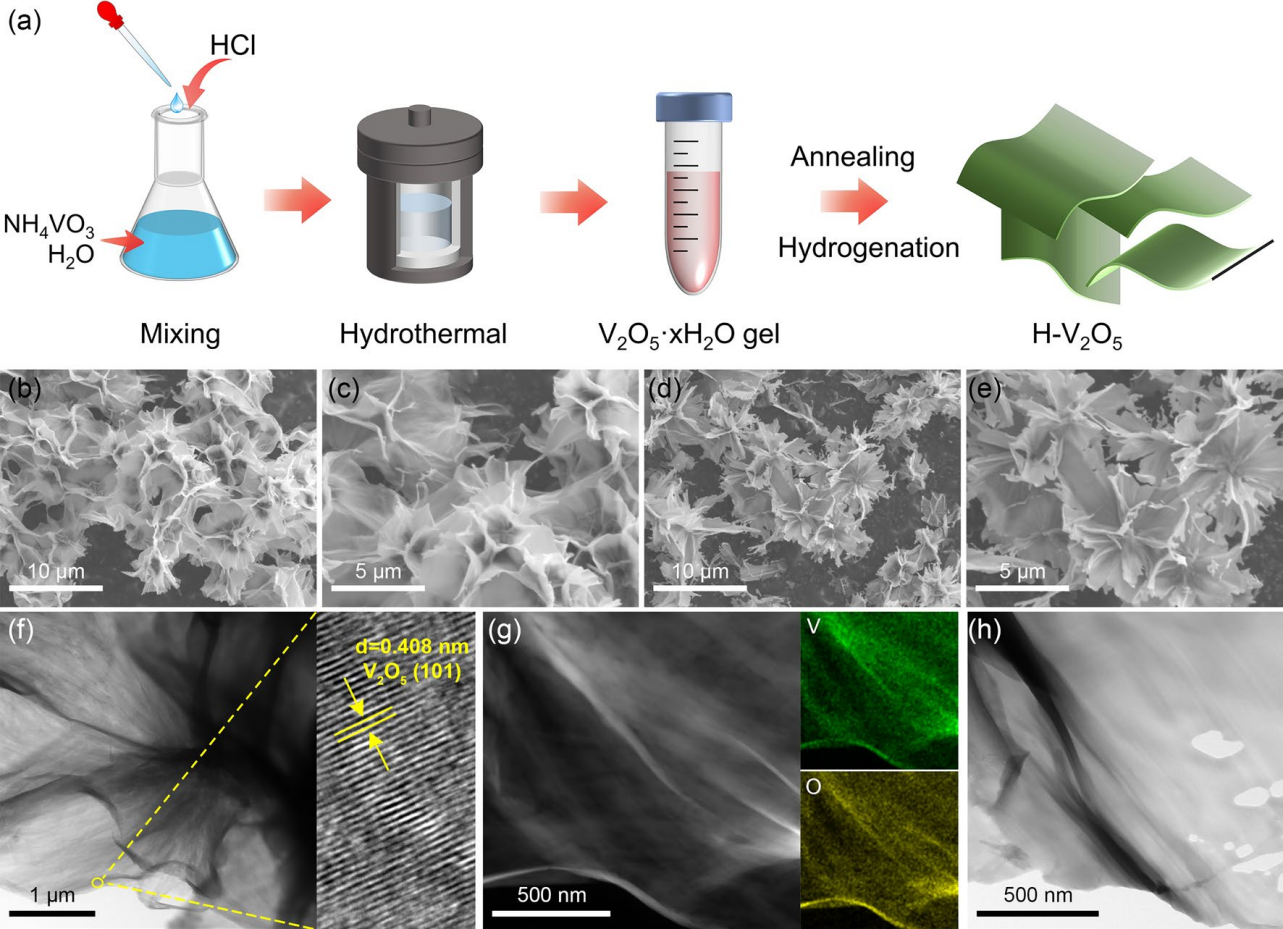
**a** Schematic illustration of the synthetic process of the H-V_2_O_5_ nanosheets. Typical SEM images of **b, c** pristine V_2_O_5_·xH_2_O and **d, e** H-V_2_O_5_ nanosheets. **f** Typical TEM and HRTEM images of pristine V_2_O_5_·xH_2_O nanosheets. **g** HAADF image and the corresponding elemental mapping of pristine V_2_O_5_·xH_2_O nanosheets. **h** Typical TEM image of H-V_2_O_5_ nanosheets

As schematically illustrated in Fig. 1a, H-V_2_O_5_ nanosheets were synthesized by a solvothermal reaction of NH_4_VO_3_ in a dilute HCl solution followed by thermal treatment under a hydrogen atmosphere. Figure S1 shows the digital photos of V_2_O_5_ nanosheets at different states. SEM images (Fig. 1b, c) validate that the as-synthesized V_2_O_5_·xH_2_O nanosheets obtained by the solvothermal reaction exhibited the interpenetrating 2D nanosheets forming a 3D nanostructure. The V_2_O_5_·xH_2_O nanosheets were then annealed in air to remove water and obtain V_2_O_5_-350air nanosheets. After hydrogenation via thermal treatment under a hydrogen atmosphere at 300 °C (H-V_2_O_5_), the morphology of the interpenetrating H-V_2_O_5_ nanosheets was perfectly retained as shown in Fig. 1d, e. Impressively, the H-V_2_O_5_ nanosheets obtained by hydrogenation of V_2_O_5_-350air nanosheets at different temperatures exhibited different colors ranging from brown to black, in marked contrast with the orange V_2_O_5_-350air powders (Fig. S1). The color change of V_2_O_5_ is ascribed to the presence of oxygen vacancies, which coincides well with the previous work [41].

**Fig. 2:**
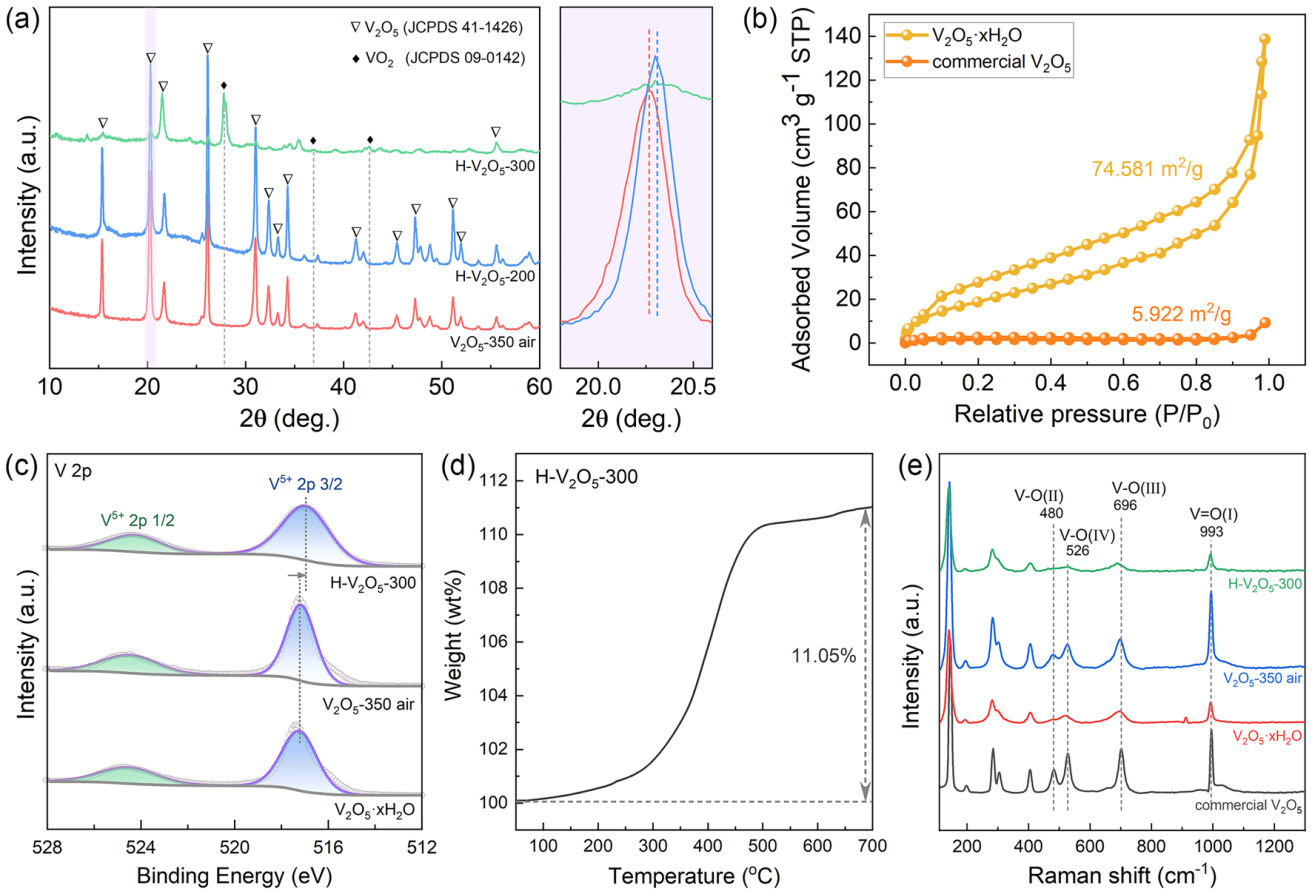
**a** XRD patterns of V_2_O_5_ nanosheets with different states. (The enlarged image shows the peak shift observed from different samples.) **b** N_2_ ad/de-sorption isotherms and corresponding specific surface areas of V_2_O_5_·xH_2_O and commercial V_2_O_5_. **c** High-resolution V 2*p* XPS spectra acquired from the V_2_O_5_·xH_2_O, V_2_O_5_-350air, and H-V_2_O_5_-300. **d** TG curve of H-V_2_O_5_-300 in air. **e** Raman spectra obtained from different samples

TEM image of pristine V_2_O_5_·xH_2_O nanosheets reveals an intertwined 2D nanosheet structure with a smooth surface (Fig. 1f). High-resolution TEM (HRTEM) image exhibits the lattice fringes of 0.408 nm (Fig. 1f), corresponding to the (101) plane of V_2_O_5_, which is in good agreement with XRD and XPS results (Fig. 2). Energy-dispersive spectroscopy (EDS) elemental mapping results demonstrate the uniform distribution of V, O, and N elements in the as-synthesized V_2_O_5_·xH_2_O nanosheets (Figs. 1g and S2). The electron-donating ability of N atoms could enhance the charge transfer, facilitating the decomposition of hydrogen molecule, and destabilization of the Mg-H bond [42, 43]. However, due to the small content of N, the effect of N element on the improvement of properties is not emphasized in this work. The TEM image of H-V_2_O_5_ nanosheets (Fig. 1h) reveals a similar morphology as the pristine V_2_O_5_·xH_2_O nanosheets. The thin 2D nanosheets provide a larger contact area with MgH_2_, thereby shortening the path enabling fast H diffusion and electron transport during the de/re-hydrogenation, giving rise to an improved hydrogen storage performance of MgH_2_.

XRD patterns of the hydrothermal product are depicted in Fig. S3, revealing peaks corresponding to the (001), (003), (005), and (006) planes of the layered V_2_O_5_·xH_2_O [44]. The V_2_O_5_-350air was obtained after annealing in air at 350 °C to remove absorbed water, and the diffraction peaks can be indexed to the orthorhombic phase V_2_O_5_ (JCPDS No. 41-1426) (Fig. S3). Furthermore, Fig. 2a shows the XRD patterns of H-V_2_O_5_ annealed at different temperatures to investigate the effects of hydrogenation temperature on the crystal structure of V_2_O_5_. It can be seen that H-V_2_O_5_-200 shows the same orthorhombic phase as V_2_O_5_-350air. However, when the hydrogenation temperature is increased to 300 °C, the characteristic diffraction peaks of the monoclinic VO_2_ phase (JCPDS No. 09-0142) can be detected, indicating that oxygen is partially removed from V_2_O_5_ at higher temperatures, which coincides well with the color change of digital photos of V_2_O_5_ nanosheets at different states (Fig. S1). The enlarged image in Fig. 2a reveals that the (001) diffraction peak of the H-V_2_O_5_-200 shifts to a higher angle, suggesting the decrease in the *d* space and the presence of oxygen vacancies in the crystal [45–47]. The specific surface areas of V_2_O_5_ nanosheets were further investigated (Fig. 2b). Compared with commercial V_2_O_5_, the specific surface area of V_2_O_5_·xH_2_O nanosheets was increased by 13 times, attributed to the special intertwined 2D nanosheet structure.

Figure 2c shows the XPS spectrum of V 2*p* in V_2_O_5_ nanosheets, which can be well split into two peaks of V at 517.1 and 524.6 eV, ascribing to the V 2*p*_3/2_ and 2*p*_1/2_ of V^5+^, respectively. Compared to the V_2_O_5_·xH_2_O and V_2_O_5_-350air, the slight shift of the V^5+^ peak of H-V_2_O_5_-300 was observed, which could be attributed to the existence of oxygen vacancy. Moreover, we performed TG tests (Fig. 2d) on the hydrogenated H-V_2_O_5_ nanosheets and found that there was a significant weight gain when samples were heated in an oxygen atmosphere. Assuming the chemical formula of H-V_2_O_5_-300 is V_2_O_5-x_, the value of x was calculated as 1.13 according to the TG results, corroborating the presence of oxygen vacancies in the H-V_2_O_5_-300 nanosheets. Raman spectra (Fig. 2e) were obtained to investigate the oxygen vacancy sites due to different oxygen sites in the crystal having different bond lengths with V. The peaks at 993, 696, and 526 cm^−1^ were assigned to the stretching vibration of V = O(I), V–O(III), and V–O(IV) bonds, respectively [48, 49]. The typical Raman peaks at 480 and 303 cm^−1^ were attributed to the bending vibrations of V–O(II) and V–O(IV) bonds, whereas those at 406 and 283 cm^−1^ correspond to the bending vibration of the V = O(I) bonds. The two Raman bands at 194 and 141 cm^−1^ were assigned to [VO_5_]-[VO_5_] vibrations [50–52]. After hydrogenation at 300 °C, the peak at 480 cm^−1^ stemming from the bending vibration of the V–O(II) bridge was almost absent, revealing that the oxygen vacancy site in H-V_2_O_5_ nanosheets is mainly at the bridging O(II) sites. Notably, hydrogenation treatment of V_2_O_5_ nanosheets can kill two birds with one stone, which not only can introduce oxygen vacancies into the oxide, but also is equivalent to a pre-treatment of the catalyst, ensuring the stability of V_2_O_5_ in the subsequent hydrogen ab/de-sorption test.

### Catalytic Effect of H-V_2_O_5_ Nanosheets on MgH_2_

**Fig. 3:**
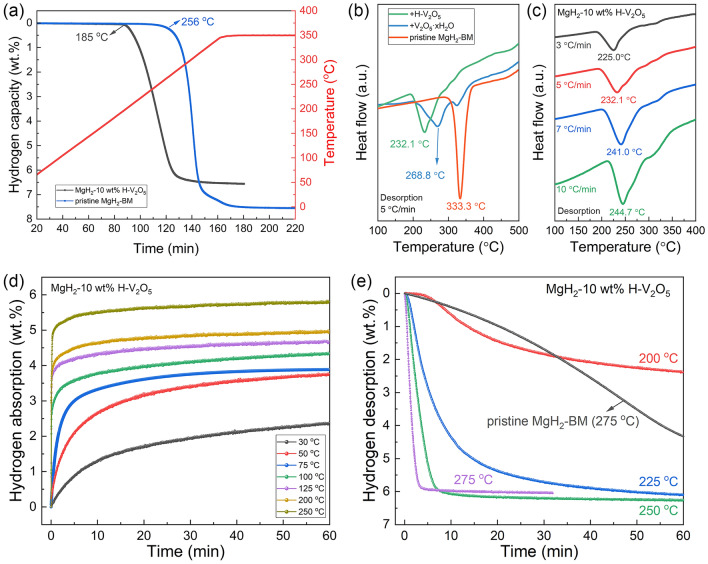
**a** TPD results of H-V_2_O_5_-doped MgH_2_ samples and pristine MgH_2_-BM. **b** DSC curves of MgH_2_ doped with different catalysts and **c** MgH_2_-10 wt% H-V_2_O_5_ with different heating rates. **d** Isothermal H_2_ absorption curves of MgH_2_-10 wt% H-V_2_O_5_ at various temperatures. **e** Isothermal H_2_ desorption curves of MgH_2_-10 wt% H-V_2_O_5_ at various temperatures (including the desorption curves of pristine MgH_2_-BM at 275 °C for comparison)

The catalytic effect of V_2_O_5_ nanosheets in improving hydrogen storage performances of MgH_2_ is evaluated by mixing V_2_O_5_ nanosheets with MgH_2_ through mechanical ball-milling. The temperature-programed desorption of H-V_2_O_5_-doped samples and pristine MgH_2_-BM were first tested. As shown in Fig. 3a, the pristine MgH_2_-BM starts releasing H_2_ at approximately 256 °C with a high terminal temperature at 350 °C, delivering a hydrogen desorption capacity of about 7.51 wt%, which agrees well with its theoretical hydrogen capacity. The results showed that the hydrogen release rate of the H-V_2_O_5_-doped samples was significantly faster than that of the pristine MgH_2_-BM samples, especially at the beginning of the hydrogen desorption. The MgH_2_-10 wt% H-V_2_O_5_ started to release H_2_ at about 185 °C, with a hydrogen capacity of 6.54 wt%. Notably, the obvious decrease in the onset and terminal desorption temperatures of H-V_2_O_5_-doped MgH_2_ compared to those of pristine MgH_2_-BM indicates smaller particle size induced by the encapsulation effect from sheet-like structure of H-V_2_O_5_ and uniform distribution between H-V_2_O_5_ nanosheets and MgH_2_ during ball milling process, which could be verified by the microtopography and elemental mapping results, hence promoting the catalytic effect of H-V_2_O_5_ nanosheets in improving hydrogen desorption performance of MgH_2_. To further illustrate the superior catalytic activity of H-V_2_O_5_ nanosheets with oxygen vacancies towards MgH_2_, the DSC curves of V_2_O_5_·xH_2_O-doped MgH_2_, H-V_2_O_5_-doped MgH_2_, and pristine MgH_2_ with different heating rates were compared (Figs. 3b, c, S4 and S5). The peak desorption temperatures of the H-V_2_O_5_-doped MgH_2_ decreased by about 36.7 and 101.2 °C compared to that of the V_2_O_5_·xH_2_O-doped MgH_2_ and pristine MgH_2_, respectively, demonstrating the superior catalytic effect of H-V_2_O_5_ nanosheets composed of both V^5+^, V^4+^ and oxygen vacancies than V_2_O_5_·xH_2_O nanosheets in improving the hydrogen desorption properties of MgH_2_. According to the Kissinger's method [53, 54], the apparent activation energies (*E*_*a*_) of MgH_2_-10 wt% H-V_2_O_5_ (Fig. S4) and pristine MgH_2_-BM (Fig. S5) were obtained. The results showed that the *E*_*a*_ of MgH_2_-10 wt% H-V_2_O_5_ and pristine MgH_2_-BM were 113.8 and 137.4 kJ mol^−1^, respectively. Thus, the addition of H-V_2_O_5_ nanosheets can significantly reduce the apparent activation energy of dehydrogenation for MgH_2_ and improve the kinetics of the composites.

In order to comprehensively evaluate the hydrogen storage performance of MgH_2_-H-V_2_O_5_ composites, isothermal hydrogen ab/de-sorption kinetics of MgH_2_ under the catalysis of H-V_2_O_5_ nanosheets are subsequently investigated (Fig. 3d, e). In contrast, the kinetic properties of pristine MgH_2_-BM were also tested (Fig. S6). According to the results of the isothermal hydrogenation, it can be seen that MgH_2_-H-V_2_O_5_ composites exhibit excellent hydrogen absorption capability at near room temperature. Under the conditions of 30 °C and 3 MPa hydrogen pressure, the dehydrogenated MgH_2_-H-V_2_O_5_ composites could absorb 2.38 wt% in 60 min, which meet the requirement of the practical application of hydrogen storage systems. In addition to the significant hydrogen absorption properties at room temperature, the composites show superior hydrogen storage kinetics by increasing the operating temperature. As depicted in Fig. 3d, the MgH_2_-H-V_2_O_5_ composites could absorb 4.1 wt% H_2_ within 30 min at 100 °C, while pristine MgH_2_-BM showed no obvious hydrogen absorption behavior under the same conditions. Additionally, the hydrogen absorption of 4.16 wt% at 200 °C and 5.08 wt% H_2_ at 250 °C only takes 60 s, while it takes 11 min for pristine MgH_2_-BM to absorb the same amount of H_2_ at 200 °C (Fig. S6).

The isothermal dehydrogenation performances of MgH_2_-H-V_2_O_5_ composites were also measured, and the results are shown in Fig. 3e. It is shown that only 5 min is required for the complete dehydrogenation of MgH_2_-H-V_2_O_5_ with a hydrogen desorption capacity of 6.0 wt% at 275 °C. However, pristine MgH_2_-BM can only release 0.2 wt% H_2_ in 5 min and 4.2 wt% H_2_ in 60 min at 275 °C, which exhibited more sluggish kinetics than MgH_2_-H-V_2_O_5_ composites. According to the slope of the desorption curve at 275 °C, the H_2_ desorption rate of MgH_2_-H-V_2_O_5_ was calculated to be 2.3 wt% min^−1^, which is much faster than that of pristine MgH_2_-BM. At the temperature of 225 °C, 4.42 wt% H_2_ could be released from MgH_2_-H-V_2_O_5_ within 10 min, which is equivalent to 68% of the theoretical hydrogen storage capacity. More impressively, even at the lower temperature of 200 °C, about 2.37 wt% H_2_ could be desorbed from MgH_2_ under the catalysis of H-V_2_O_5_ nanosheets within 60 min.

It is worth noting that different activation energies will be obtained when different kinetic models are used for fitting. Therefore, to obtain more accurate activation energy values, an in-depth analysis of de/re-hydrogenation kinetic mechanism model of composites was carried out according to nine different kinetic models proposed by Sharp and Jones [55], and the activation energy was fitted according to a suitable kinetic model. The specific methods are provided bellow:

The general dynamic equation is shown as follows:

1 $$ {\text{d}}\upalpha /{\text{dt}} = {\text{k}}\left( {\text{T}} \right){\text{g}}\left( \upalpha \right) $$

2 $$ {\text{g}}\left( \upalpha \right) = {\text{A}}\left( {{\text{t}}/{\text{t}}_{0.5} } \right) $$

where *α*, *T*, *k*(T), *g*(α), *A*, and *t*_0.5_ represent the reaction extent, the reaction temperature, the reaction rate constant, the function depending on the specific kinetic mechanism, the constant related to the kinetic mechanism, and the time when α equals 0.5 respectively. Through plotting the dehydrogenation experimental data of t/t_0.5_ against the theoretical ones of composites for nine different kinetic models, respectively, the corresponding reliable kinetic model can be obtained (the line with a slope close to 1 is the reliable kinetic model).

**Fig. 4:**
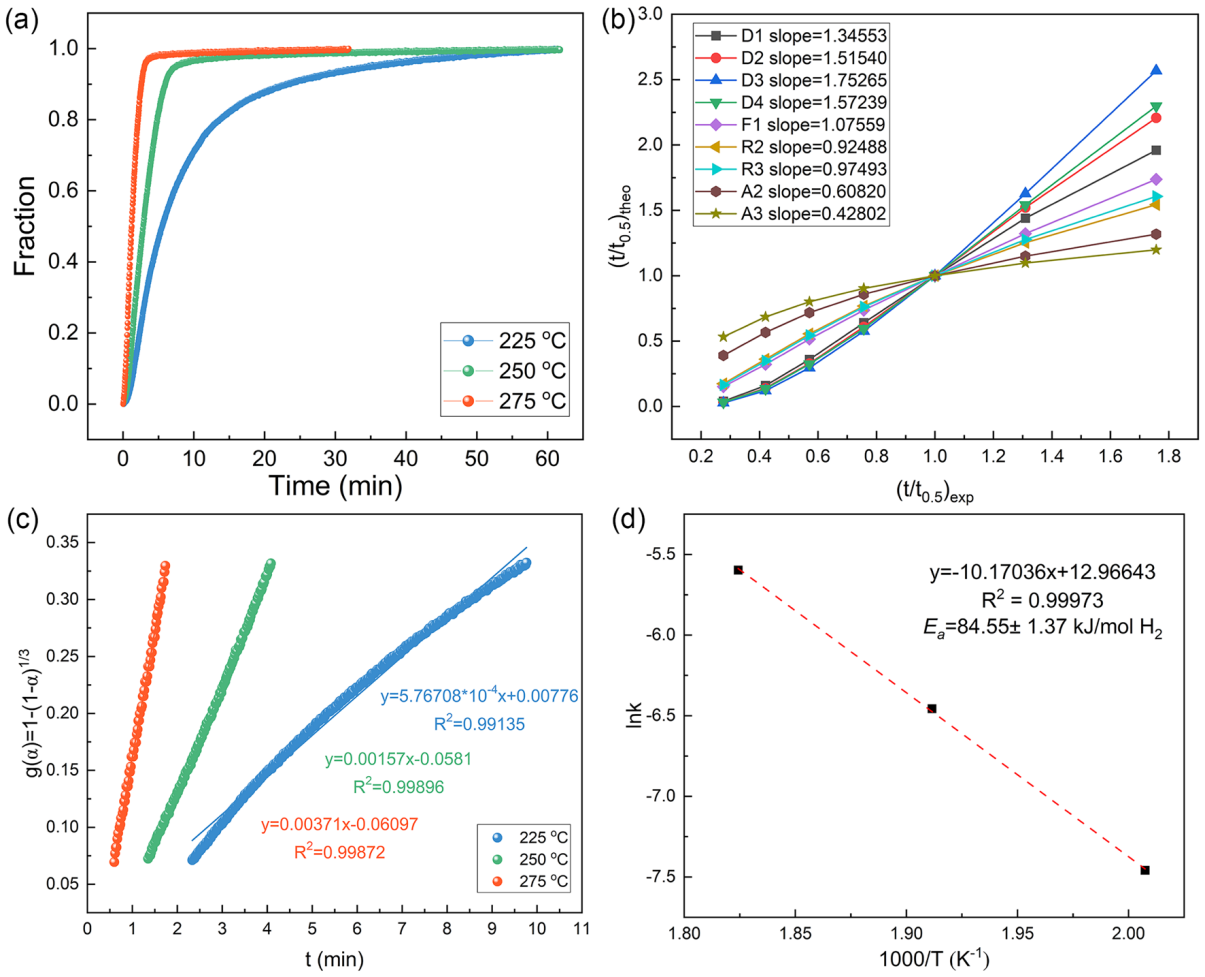
**a** The extent of reaction curves of MgH_2_-10 wt% H-V_2_O_5_ composites at 225, 250, and 275 °C. **b** (t/t_0.5_)_theo_ vs. (t/t_0.5_)_exp_ of composites at 225 °C for various kinetic models. **c** Time dependence of kinetic modeling equations g(α) for composites with 0.2 < α < 0.7 at different temperatures. **d** Calculation of the apparent activation energies according to the Arrhenius equation

As shown in Fig. 4b, we obtained nine curves based on the isothermal dehydrogenation curves of composites at 225 °C. In our case, the dehydrogenation kinetic model should be R3 model, which has the slope value of 0.97493 close to 1. The R3 model is further verified through plotting the *g*(α) related to R3 against the reaction time at 250 and 275 °C when α ranges from 0.2 to 0.7, which exhibit strong linear relationship (R^2^ more than 0.99) as shown in Fig. 4c. Moreover, the apparent activation energy (*E*_*a*_) was calculated to evaluate the energy barriers for the dehydrogenation process of MgH_2_-10 wt% H-V_2_O_5_ according to the following Arrhenius equation:

3 $$ {\text{lnk}} = - {\text{E}}_{{\text{a}}} /{\text{RT}} + \ln {\text{A}} $$

where R and *k* represent gas and rate constant, respectively. And *k* is the slope value that can be extracted from Fig. 4c. Through linear fitting the lnk and 1000/T [53, 56], the *E*_*a*_ (84.55 ± 1.37 kJ mol^−1^ H_2_) for dehydrogenation was obtained as shown in Fig. 4d. Similarly, we have re-fitted the kinetic model of the hydrogen absorption process as well as the hydrogen absorption activation energy barrier according to the same method, and the results are shown in Fig. S7. According to the calculation results, we get the precise hydrogen absorption activation energy *E*_*a*_ (42.55 ± 0.41 kJ mol^−1^ H_2_). The *E*_*a*_ values for ab/de-sorption of MgH_2_-H-V_2_O_5_ were significantly lower than those of the commercial MgH_2_ (71 kJ mol^−1^ H_2_ for absorption and 160 kJ mol^−1^ H_2_ for desorption) [9, 54]. Overall, the introduction of H-V_2_O_5_ nanosheets greatly improved the kinetic performances of MgH_2_.

The thermodynamic properties of the MgH_2_-H-V_2_O_5_ composites and pristine MgH_2_-BM were determined by the PCI curve (Fig. S8). According to the van’t Hoff plots, the de/re-hydrogenation enthalpy (*ΔH*) of the composites were determined to be 73.4 and − 73.6 kJ mol^−1^ H_2_ (Fig. S8a, b), respectively, which were comparable to the *ΔH* of pristine MgH_2_-BM (Fig. S8c, d). It demonstrates that the introduction of H-V_2_O_5_ nanosheets exhibits no effect in tuning the thermodynamics of MgH_2_, confirming that the modification of hydrogen storage properties of MgH_2_ by H-V_2_O_5_ nanosheets is mainly a catalytic effect. Notably, compared to pristine MgH_2_-BM, the platform pressure of MgH_2_-H-V_2_O_5_ composites obviously increased, which was closer to the requirements of commercial applications.

**Fig. 5:**
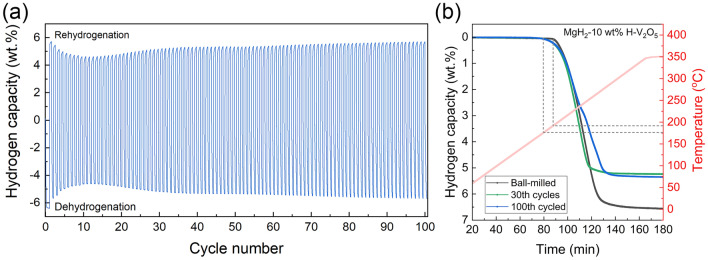
**a** De/re-hydrogenation cycle curves of MgH_2_-10 wt% H-V_2_O_5_. **b** TPD results of MgH_2_-10 wt% H-V_2_O_5_ upon cycling

**Fig. 6:**
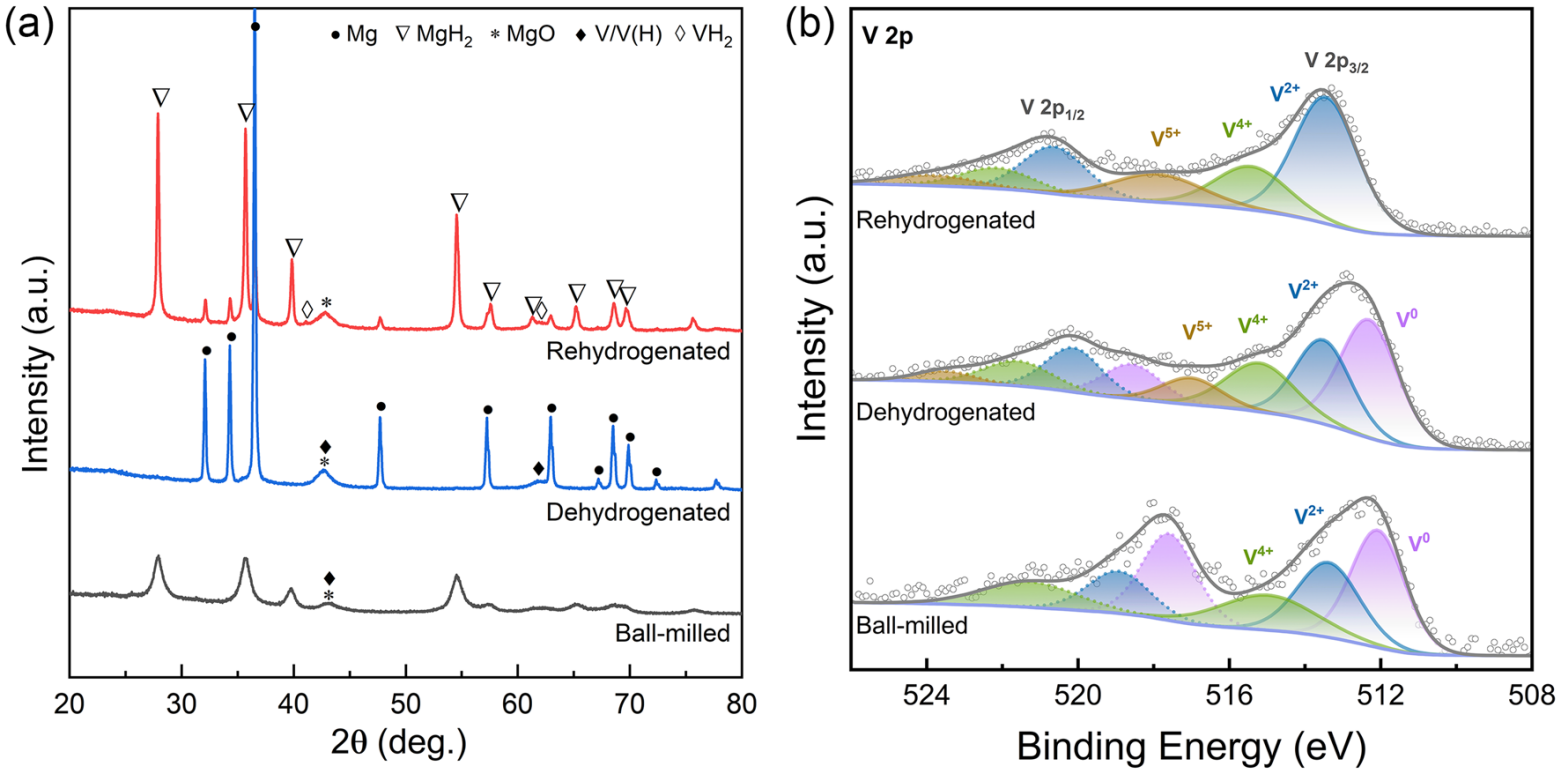
**a** XRD patterns and **b** high-resolution V 2*p* XPS spectra of MgH_2_ under the catalysis of H-V_2_O_5_ nanosheets at various states

The hydrogen ab/de-sorption cycling curves of the MgH_2_-H-V_2_O_5_ sample at 275 °C are shown in Figs. 5 and S9. The composites could achieve the reversible de/re-hydrogenation with the hydrogen capacity of about 5.7 wt% during the first cycle. As the number of cycles increased, the hydrogenation capacity of the composites decreased slightly compared with that of the first cycle, which may be due to the formation of a surface passivation oxide layer on the MgH_2_/Mg particles and incomplete activation during the first few cycles, limiting the hydrogen uptake/release capacity. Additionally, the H-V_2_O_5_ catalyst may undergo a reduction process during the initial cycles, which may contribute to the decrease in capacity. Moreover, the metal V generated during the ball milling process could absorb hydrogen to form VH_2_ species, which was proved by the presence of the V^2+^ in the XPS results of dehydrogenated samples (Fig. 6b). Due to the fact that V-H bonds are very stable, VH_2_ is difficult to be completely dehydrogenated, resulting in a decrease in the hydrogen storage capacity. However, after 10 cycles, the capacity attenuation tended to be stable. More impressively, as the hydrogen ab/de-sorption cycle continued, the capacity of the composites started to recover and slowly increased, gradually returning to the capacity of the first hydrogen absorption cycle. As the cycling continued, the surface oxide layer can gradually be broken down, creating a highly homogeneous and intimate contact between MgH_2_ and H-V_2_O_5_ and forming a stable structure. Additionally, the presence of oxygen vacancies may inspire the decomposition of VH_2_ and promote the transformation between V(H) and VH_2_, thus, contributing a portion of the hydrogen storage capacity, which is proved later in the results of the theoretical calculations. The H-V_2_O_5_ catalyst may also undergo a gradual activation process, restoring its catalytic activity and enhancing the hydrogen storage performance of MgH_2_/Mg. Furthermore, the cycling-induced changes in the electronic structure of the H-V_2_O_5_ catalyst introduced the multi-valance V into composites, which can also contribute to the improved kinetics and stable cyclic behavior. After even 100 cycles, the hydrogen capacity of the composite was still maintained at about 5.7 wt%, which was remarkably better than those of the state-of-the-art V_2_O_5_-catalyzed MgH_2_ systems. Regarding the amount of hydrogen absorption in the first cycle as a benchmark, the composites could maintain a higher capacity retention rate of 99% after 100 cycles. In contrast, the capacity of pristine MgH_2_-BM without catalysts decreased significantly during the de/re-hydrogenation cycles [25, 57].

More interestingly, a decline of the onset temperature from 185 to 175 °C could be clearly observed for MgH_2_ under the catalysis of H-V_2_O_5_ nanosheets since the 30th cycle of hydrogen desorption process (Fig. 5b), indicating the enhancement of the catalytic effect of H-V_2_O_5_ nanosheets upon cycling process. Even after 100 cycles, the initial hydrogen desorption temperature and hydrogen capacity of MgH_2_-H-V_2_O_5_ composites showed no obvious attenuation. The re-hydrogenated composites could desorb hydrogen at lower temperature ranges than that of the ball-milled samples, indicating that the chemical states of H-V_2_O_5_ nanosheets might slightly change and the catalytic activity was significantly improved during the de/re-hydrogenation cycling behavior. By comparison, the onset dehydrogenation temperature of pristine MgH_2_-BM increased significantly from 256 to 290 °C after 20 cycles, showing a significant decay of performance (Fig. S10). This indicates that the addition of H-V_2_O_5_ nanosheets can not only provide catalytic activity for MgH_2_, but also inhibit the serve agglomeration and growth of MgH_2_ particles from the unique sheets-like structure of H-V_2_O_5_ nanosheets, thereby improving the cycling stability of the MgH_2_-H-V_2_O_5_ composites. Notably, as the test temperature increases, the large MgH_2_ particles continue to release hydrogen at high temperatures, and the maximum hydrogen storage capacity of the MgH_2_-BM does not decay (Fig. S10). In contrast, the hydrogen storage capacity of the composite decreases after several hydrogen ab/de-sorption cycles, and this capacity cannot be recovered with the increase of test temperature (Fig. 5b). This further proves that the attenuation of capacity is not caused by the particle growth, but by the formation of the new catalytic phase.

### Understanding the Synergistic Catalytic Mechanism

To elucidate the catalytic mechanism of the H-V_2_O_5_ nanosheets for MgH_2_, a series of characterizations were carefully performed to study the corresponding structures and compositions of the MgH_2_-H-V_2_O_5_ at different states. The XRD results of MgH_2_-H-V_2_O_5_ before and after de/re-hydrogenation are shown together with the pristine MgH_2_ after ball milling in Figs. 6 and S11. The XRD patterns show that the β-MgH_2_ with a little γ-MgH_2_ is the main phases in the pristine MgH_2_ after ball milling (Fig. S11), indicating the high purity of the MgH_2_ powder. In addition to the peak of MgH_2_, a small amount of V phase and MgO can be detected in the XRD patterns of the ball-milled MgH_2_-H-V_2_O_5_ composites (Fig. 6a) and these two phases were difficult to distinguish due to their overlapping Bragg reflections, demonstrating the partial reduction of H-V_2_O_5_ by MgH_2_ during the ball milling process (5MgH_2_ + V_2_O_5_ = 5MgO + 2V + 5H_2_, ΔG = − 1246 kJ mol^−1^ V_2_O_5_). The absence of MgO phase in pristine MgH_2_-BM further indicates that H-V_2_O_5_ and MgH_2_ undergo a redox reaction during the ball milling process. This reaction in-situ generates fine, dispersed, and catalytically active metal V along with inert MgO, consuming a small amount of Mg, thus, providing evidence for the decay of the capacity in the initial cycling test in the following section. Additionally, previous studies have shown that metal V could serve as hydrogen spillover active sites to promote the dissociation of hydrogen molecules [24, 29, 58]. After dehydrogenation, the peaks from MgH_2_ completely disappeared, and the XRD characteristic peaks of Mg and V appeared. Notably, Mg phase was converted to MgH_2_ accompanied by the appearance of VH_2_ in the subsequent hydrogen absorption process. Based on the above results, we can deduce that part of H-V_2_O_5_ is reduced to the V phase, which can then transform to VH_2_ after re-hydrogenation. The reversible chemical reaction between V/V(H) and VH_2_ plays a crucial role in the catalytic effect of H-V_2_O_5_ on MgH_2_ [29, 59], which is evidenced in the XPS and HRTEM analysis. The XRD patterns of the MgH_2_-H-V_2_O_5_ composites after 100 re/de-hydrogenation cycles were also depicted in Figs. S12 and S13. After 100 cycles, the main characteristic diffraction peaks of MgH_2_/Mg with the catalysis of H-V_2_O_5_ nanosheets could be indexed to MgH_2_/Mg, and V/VH_2_ phase can also be detected, which provides additional evidence for the high reversibility of MgH_2_ and stable catalytic activity of V/VH_2_.

XPS analysis and HRTEM measurement were further conducted to analyze the state changes of V in the MgH_2_-H-V_2_O_5_ composites. After the ball milling process, the spin–orbit double peaks at 512.08/517.6 eV, 513.38/518.93 eV, and 514.98/521.28 eV in the V 2*p* XPS spectra (Fig. 6b) could be indexed to V^0^, V^2+^, and V^4+^, respectively, indicating that part of the high-valence V^5+^ ions were reduced to metallic V^0^. The existence of V^4+^ evidenced the presence of oxygen vacancies. It is worth emphasizing that the absence of the signal of V^5+^ may be due to the fact that the detection depth of XPS is only a few nanometers below the surface, while the H-V_2_O_5_ nanosheets may be covered by MgH_2_ or partially reduced metallic V nanoparticles in the ball milling process and undetectable by the XPS measurement. After the subsequent activation process of hydrogen ab/de-sorption, the H-V_2_O_5_ nanosheets were gradually exposed to the surface, which was further proved by the detection of the signal of V^5+^ in the XPS spectra of the de/re-hydrogenated samples. The combined analysis of XPS and XRD results showed that a phase transition between VH_2_ and V/V(H) occurred during the hydrogen ab/de-sorption process. Notably, the presence of V^2+^ in the dehydrogenated samples indicated that VH_2_ was not completely dehydrogenated, which further provided evidence for the capacity decay at the beginning of the cycle. Moreover, compared to the ball-milled samples, the slightly shift of V^0^ peak of the dehydrogenated samples was observed due to the incomplete dehydrogenation of VH_2_ and the existence of V(H). Impressively, after 100 cycles, the absence of V^2+^ in the dehydrogenated sample indicated that VH_2_ was activated as an active catalyst and completely dehydrogenated to produce V/V(H), attributed to the pre-activation process and the assistance of oxygen vacancies.

The results further indicate that in-situ formed V could facilitate the spontaneous H_2_ dissociation, stemming from the strong hybridization between the molecular orbital of adsorbed hydrogen and the V 3*d* states [60], which could promote hydrogen spillover from the V/H-V_2_O_5_ multi-phase catalysts to the Mg surface. Moreover, computational study [61] showed that the formation energy of the VH_2_/MgH_2_ interface had a low absolute value of the negative heat compared with that of MgH_2_, demonstrating that VH_2_/V enhanced the desorption properties of MgH_2_. Hence, it could be concluded that the hydrogen spillover effect originated from V/H-V_2_O_5_ catalysts and rapid electron transfer between multi-valent V states facilitate the hydrogen ab/de-sorption process of MgH_2_. Furthermore, the high-resolution O 1* s* XPS spectra (Fig. S14) demonstrated the presence of oxygen vacancy and it remains stable in the process of re/de-hydrogenation. Notably, it is deemed that the bonding energy between V and H atoms is strong. Hence, we deduced that oxygen vacancy might stimulate the phase transition between VH_2_ and V, which was further proved by theoretical calculation in the next section.

**Fig. 7:**
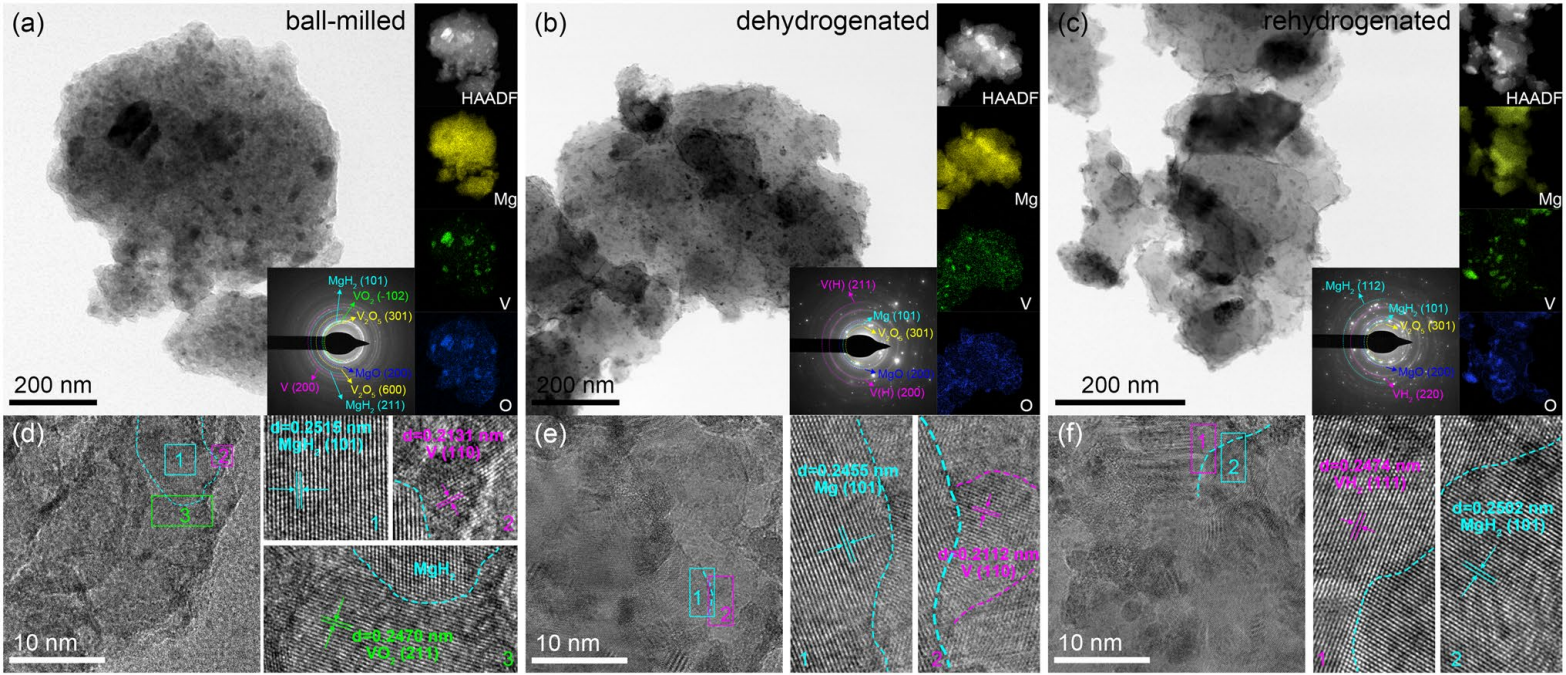
**a**–**c** The typical bright field TEM images, the HAADF images, the corresponding elemental mapping, and **d**–**f** the relative HRTEM images of MgH_2_-H-V_2_O_5_ composites at various states (The inset in **a**–**c** shows the corresponding SAED patterns)

Furthermore, the morphologies of MgH_2_-H-V_2_O_5_ in the three different states were presented to clarify the evolution of the microstructure of the composites (Fig. 7). The dark regions with lamellar distribution in the BF-TEM images could be identified as the H-V_2_O_5_ nanosheets, and the well-distributed dark regions in the form of dots indicate V/V(H)/VH_2_ nanoparticles from the reduction of H-V_2_O_5_ nanosheets during ball milling process. It can be seen that the sheet-like structure of H-V_2_O_5_ still maintained well after re/de-hydrogenation, encapsulating around the surface of MgH_2_ nanoparticles, which inhibited the agglomeration and growth of MgH_2_ to a certain extent and ensured its excellent cycling stability. It is worth noting that the morphology of MgH_2_ nanoparticles remained stable after the hydrogen ab/de-sorption cycling tests (Fig. S15). However, the pristine MgH_2_-BM sample without catalyst doping exhibited obvious agglomeration after re/de-hydrogenation cycles (Fig. S16), which coincided well with the poorly tested cyclic stability of pristine MgH_2_. Additionally, the elemental mapping of MgH_2_-H-V_2_O_5_ in different states illustrated that Mg, V, and O elements were homogeneously distributed in the composites.

From the HRTEM characterizations of ball-milled composites, it was observed that the spacing values of 0.2515 and 0.2470 nm matched well with those of MgH_2_ (101) and VO_2_ (211) (Fig. 7d) in MgH_2_-H-V_2_O_5_, respectively. The lattice spacing of 0.2131 nm could be indexed to the (101) planes of metallic V. In the dehydrogenation states, the spacing value of 0.2455 nm corresponded to the Mg (101) and the metallic V distributing around the Mg phase could still be detected. (Fig. 7e). For the rehydrogenated samples, the spacing values of 0.2502 and 0.2474 nm were indexed to MgH_2_ (101) and VH_2_ (111), respectively (Fig. 7f), indicating that V nanoparticles formed by the reduction of H-V_2_O_5_ could transform to VH_2_ during the rehydrogenation process.

To further quantitatively understand the synergistic catalytic effect of V_2_O_5_ and oxygen vacancies, especially in the VH_2_/H-V_2_O_5_ interface in the hydrogen storage properties of MgH_2_, DFT calculations were performed within MgH_2_ (110), Mg (100), V_2_O_5_ (001), and VH_2_ (111) surface model. According to the calculation results, introducing V_2_O_5_ into the MgH_2_ exhibited a positive effect on decreasing the de/re-hydrogenation energy barriers of MgH_2_/Mg and destabilizing the Mg-H bonds. Moreover, the introduction of oxygen vacancies will further enhance this effect. As shown in Fig. S17 and Table S1, the length of the Mg-H bond of pristine MgH_2_ was 1.72 Å. After the addition of V_2_O_5_, the Mg-H bond extended to 1.79 Å. Notably, obvious elongation of the Mg-H bond to 1.88 Å was obtained by further introducing the oxygen vacancies into the system. Furthermore, the bond energy of the Mg-H was also reduced from 92.39 kJ mol^−1^ for pure MgH_2_ to 62.36 and 40.93 kJ mol^−1^ after the introduction of V_2_O_5_ nanosheets and oxygen vacancies, respectively. Increasing of the bond length and decreasing of bond energy are deemed to be the most direct evidence for the weakening of Mg-H bond, demonstrating that it is easier to break the Mg-H bond with lower desorption temperature [62].

**Fig. 8:**
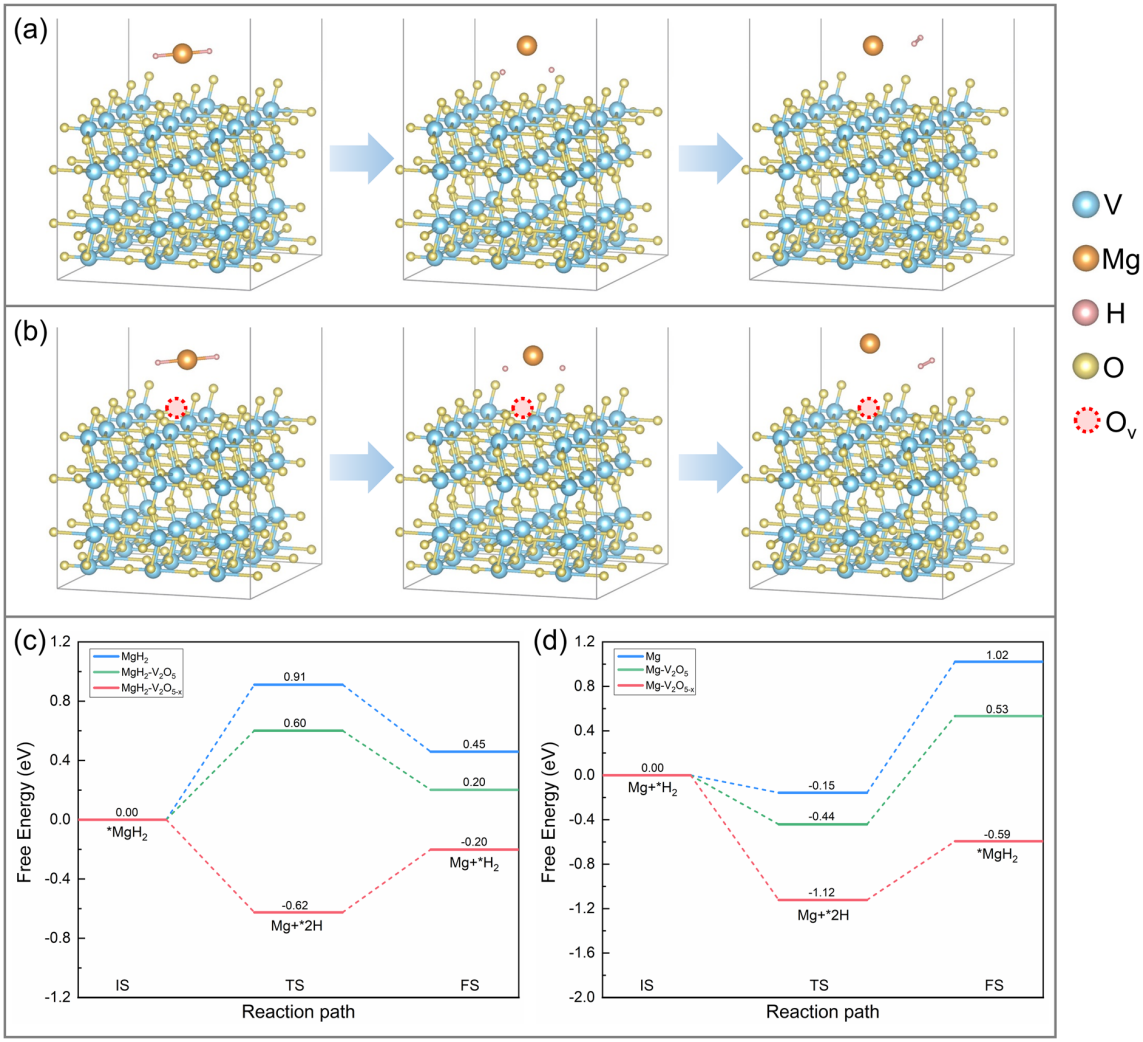
Schematic illustration of the H_2_ desorption process of **a** MgH_2_ on the V_2_O_5_ (001) plane and **b** MgH_2_ on the V_2_O_5-x_ (001) plane. Calculated energy profiles for the **c** H_2_ desorption and **d** absorption of MgH_2_

**Fig. 9:**
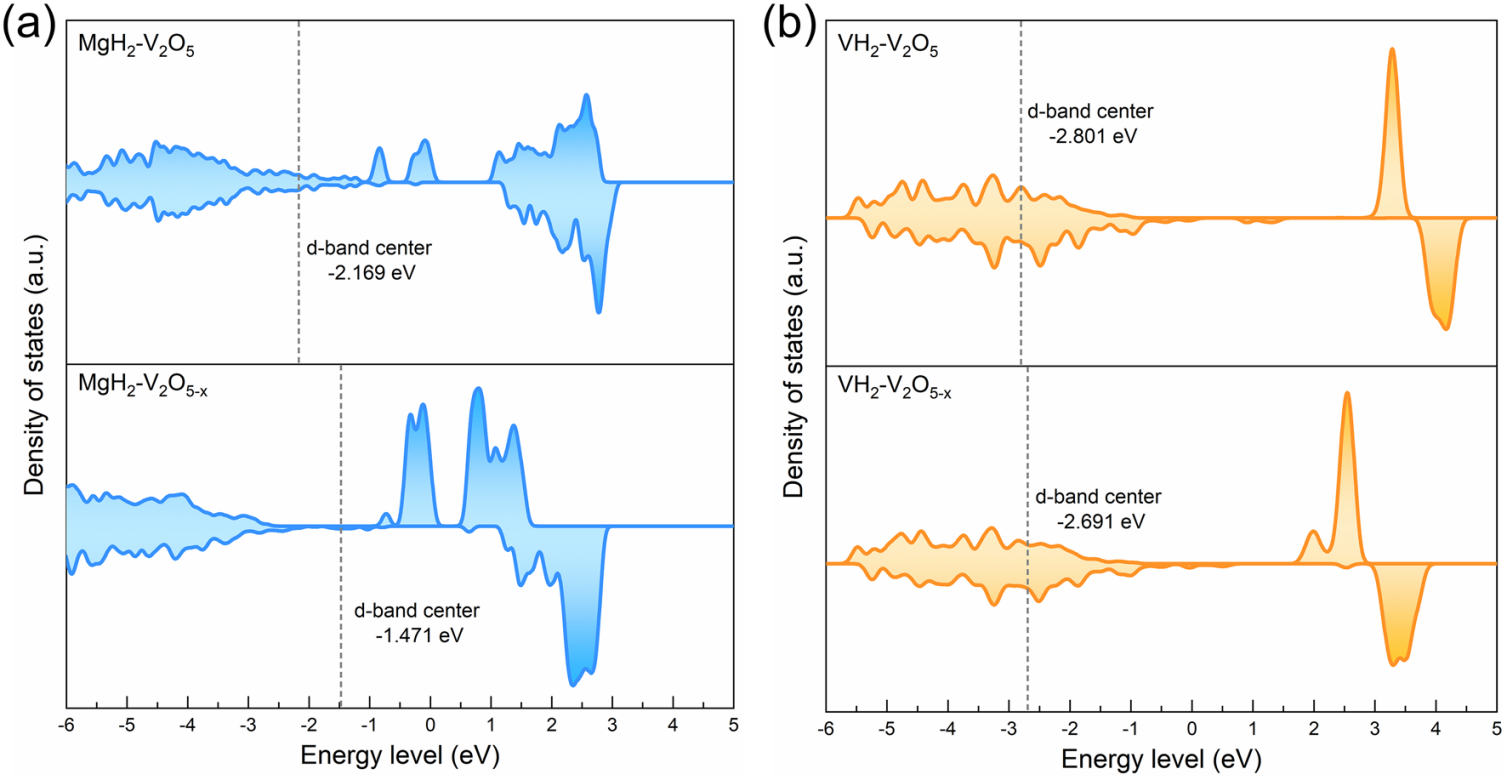
DOS of the **a** MgH_2_-V_2_O_5_/V_2_O_5-x_ system and **b** VH_2_-V_2_O_5_/V_2_O_5-x_ system

Additionally, the energy barrier for the de/re-hydrogenation process of MgH_2_/Mg on V_2_O_5_ (001) and V_2_O_5-x_ (001) are subsequently calculated, as schematically illustrated in Fig. 8. Hypothetically, the hydrogen desorption process of MgH_2_ includes two steps: MgH_2_ adsorbed on V_2_O_5_ (001)/V_2_O_5-x_ (001) substrates (initial state, denoted as IS) undergoes the breakage of Mg-H bonds (transition state, TS), and then the H atoms escape from the substrate and recombine into H_2_ (final state, FS). As shown in Fig. 8c, d, the free energy for de/re-hydrogenation of pristine MgH_2_/Mg was calculated to be 0.91/-0.15 eV. In contrast, obvious decreased energy barrier can be noticed under the catalysis of V_2_O_5_ (0.60/-0.44 eV) and oxygen vacancies (-0.62/-1.12 eV), which directly implies their superior catalytic activities in reducing the hydrogen sorption temperatures of MgH_2_. Specifically, the negative energy barrier value of the MgH_2_-V_2_O_5-x_ system demonstrates that the decomposition of MgH_2_ on oxygen vacancies-rich V_2_O_5_ nanosheets is spontaneous, implying that the dehydrogenation of MgH_2_ is further facilitated by the accelerated electron transfer due to the introduction of oxygen vacancies and this can be further proved from the DOS curves. Figure 9a shows the DOS and *d*-orbital bias density of MgH_2_-V_2_O_5_ system and MgH_2_-V_2_O_5-x_ system, it was demonstrated that the *d*-band centers of the MgH_2_-V_2_O_5-x_ system was closer to the Fermi energy level compared to the MgH_2_-V_2_O_5_ system, indicating the increase in electron distribution.

Moreover, based on the results of hydrogen storage performance test, we have concluded that the presence of oxygen vacancies may inspire the decomposition of VH_2_ and promote the transformation between V(H) and VH_2_. In order to better illuminate the inspiration function of oxygen vacancies on the decomposition of VH_2_, the length of the V-H bond of pristine VH_2_ model, VH_2_ on V_2_O_5_ (001) surface model, and VH_2_ on V_2_O_5-x_ (001) surface model were further calculated (Fig. S18). As shown in Table S2, under the catalysis of V_2_O_5_ and V_2_O_5-x_, the length of the V-H bond was significantly increased from 1.87 Å (pristine VH_2_) to 1.93 and 2.04 Å, respectively, and the bond energy was also reduced from 75.663 kJ mol^−1^ in pure (pristine VH_2_) to 70.691 and 52.613 kJ mol^−1^, which directly demonstrated the inspiration effect of oxygen vacancies on the decomposition of VH_2_. Moreover, the DOS and *d*-orbital bias density of VH_2_-V_2_O_5_ system and VH_2_-V_2_O_5-x_ system (Fig. 9b) further demonstrated that the introduction of oxygen vacancies could increase the electron distribution, thus weakening the V-H bond.

The above calculation results provide a rational explanation for lower de/ab-sorption temperatures and energy barrier in MgH_2_-H-V_2_O_5_ composites. The presence of oxygen vacancies not only effectively promotes the dissociation of MgH_2_, but also reduces the dehydrogenation energy barrier of the VH_2_ catalyst, further improving its catalytic activity. In other words, the introduction of oxygen vacancies can kill two birds with one stone, which can directly enhance the hydrogen storage performance of the matrix material (MgH_2_) and indirectly improve the overall hydrogen storage performance of the system by affecting the catalytic activity of the catalyst (VH_2_). These results confirm the synergistic catalytic effect of transition metal oxide and oxygen vacancies in improving the hydrogen storage performance of MgH_2_, which coincides well with previous researches [31].

Based on the above discussions, the reaction equations for the hydrogen storage process of MgH_2_-H-V_2_O_5_ composites are as follows:

4 $$ 5{\text{MgH}}_{2} + {\text{V}}_{2} {\text{O}}_{5} \to 5{\text{MgO}} + 2{\text{V}} + 5{\text{H}}_{2} $$

5 $$ {\text{V}}\left( {\text{H}} \right) + {\text{H}}_{2} \leftrightarrows {\text{VH}}_{2} $$

6 $$ {\text{Mg}} + {\text{H}}_{2} \leftrightarrows {\text{MgH}}_{2} $$

**Fig. 10:**
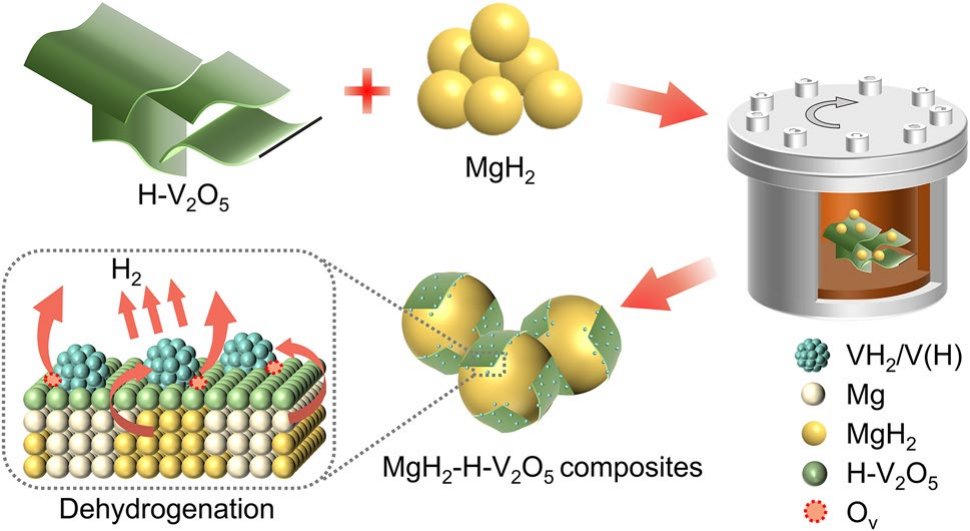
Schematic diagram showing the mechanisms of enhanced hydrogen storage performances of the MgH_2_-H-V_2_O_5_ composites

And the he possible catalytic mechanisms (Fig. 10) of H-V_2_O_5_ for the re/de-hydrogenation of MgH_2_ were proposed. (1) The sheet-like structure of the H-V_2_O_5_ with abundant oxygen vacancies was beneficial for exposing more active sites to contact with MgH_2_/Mg. The numerous interfaces between H-V_2_O_5_ nanosheets and MgH_2_/Mg provide more diffusion paths for hydrogen and nucleation sites for Mg/MgH_2_, improving its hydrogen storage performances. (2) During the ball milling process, the cross-linked 3D H-V_2_O_5_ nanosheets were broken into 2D nanosheets, which were uniformly covered on MgH_2_ surface. By virtue of the unique 2D sheet-like structure of H-V_2_O_5_, the agglomeration and growth of MgH_2_ could be inhibited through the encapsulation effect of H-V_2_O_5_, ensuring its excellent cycling stability. (3) Additionally, H-V_2_O_5_ could react with MgH_2_ during ball milling process, forming V/H-V_2_O_5_ multi-phase catalysts. Profiting from the “hydrogen spillover” effect of V/VH_2_ phase, hydrogen molecules exhibit a low dissociation energy barrier on the surface of metal V, which promotes the dissociation of H_2_ [62], facilitating the hydrogen absorption properties. (4) The presence of oxygen vacancies and multi-valent V could accelerate the electron transfer between the catalyst and Mg, thus weakening the Mg-H bond and promoting the dehydrogenation of MgH_2_ [33]. (5) Moreover, the oxygen vacancies in H-V_2_O_5_ nanosheets may inspire the "hydrogen pump" effect of VH_2_/V, promoting the dehydrogenation of VH_2_, thus further strengthen the catalytic activity of the VH_2_/V. Taken together, these results corroborate that the incorporation of hydrogenated V_2_O_5_ nanosheets with abundant oxygen vacancies leads to the improved hydrogen storage performances of MgH_2_.

## Conclusion

In conclusion, hydrogenated H-V_2_O_5_ nanosheets with abundant oxygen vacancies were successfully designed using a simple and scalable solvothermal and subsequent hydrogenation treatment method, which were then used as the catalysts to improve the hydrogen storage performance of MgH_2_. As a result, MgH_2_-H-V_2_O_5_ composites started to release H_2_ at 185 °C. Moreover, the composites can rapidly absorb 2.38 wt% H_2_ within 60 min at 30 °C and release 6.0 wt% H_2_ in 5 min at 275 °C, as well as improved desorption kinetics (*E*_*a*_ = 84.55 ± 1.37 kJ mol^−1^ H_2_), followed by long-term cyclic stability with an excellent retention rate of ~ 99% even after 100 cycles. It was confirmed that the sheet-like structure of H-V_2_O_5_, abundant oxygen vacancies, multi-valance of V species, and the formation of VH_2_/V contributed to the enhanced hydrogen storage performances of MgH_2_. Notably, the oxygen vacancies in H-V_2_O_5_ nanosheets not only directly accelerate electron transfer in de/re-hydrogenation of MgH_2_, but also inspire the “hydrogen pump” effect of VH_2_/V, promoting the catalytic activity of VH_2_/V, thus indirectly improving the hydrogen storage properties of MgH_2_. This research provides a viable path for the rational design of multi-functional oxygen vacancy-rich catalysts for MgH_2_. Furthermore, our strategy may be extended to other complex hydrides (e.g., LiBH_4_, Mg(BH_4_)_2_, NaAlH_4_…) and pave the way to explore the influence of oxygen vacancy on the performances of hydrogen storage materials.

## Supplementary Information

Below is the link to the electronic supplementary material.

Supplementary file1 (PDF 1311 KB)
