# Supplementary material for: Boosting Hydrogen Storage Performance of MgH2 by Oxygen Vacancy-Rich H-V2O5 Nanosheet as an Excited H-Pump
Source: Nanomicro Lett. 2024 Mar 21;16:160. doi: 10.1007/s40820-024-01375-8 (PMC11231122; doi:10.1007/s40820-024-01375-8)
Supplement: Supplementary file 1 — Supplementary file1 (PDF 1311 KB) [file 40820_2024_1375_MOESM1_ESM.pdf]

Supporting Information for

Boosting Hydrogen Storage Performance of MgH_2 by Oxygen Vacancy-Rich H-V_2O_5 Nanosheet as an Excited H-Pump

Li Ren [1,2,3], Yinghui Li [1,2,3], Zi Li [1,2,3], Xi Lin [1,2,3], Chong Lu [4], Wenjiang Ding [1,2,3], Jianxin Zou [1,2,3,]*

[1] National Engineering Research Center of Light Alloys Net Forming & State Key Laboratory of Metal Matrix Composites, Shanghai Jiao Tong University, Shanghai 200240, P. R. China

[2] Shanghai Engineering Research Center of Mg Materials and Applications & School of Materials Science and Engineering, Shanghai Jiao Tong University, Shanghai 200240, P. R. China

[3] Center of Hydrogen Science, Shanghai Jiao Tong University, Shanghai 200240, P. R. China

[4] Instrumental Analysis Center of SJTU, Shanghai Jiao Tong University, Shanghai 200240, P. R. China

* Corresponding author. E-mail: zoujx@sjtu.edu.cn (Jianxin Zou)

Supplementary Figures and Tables

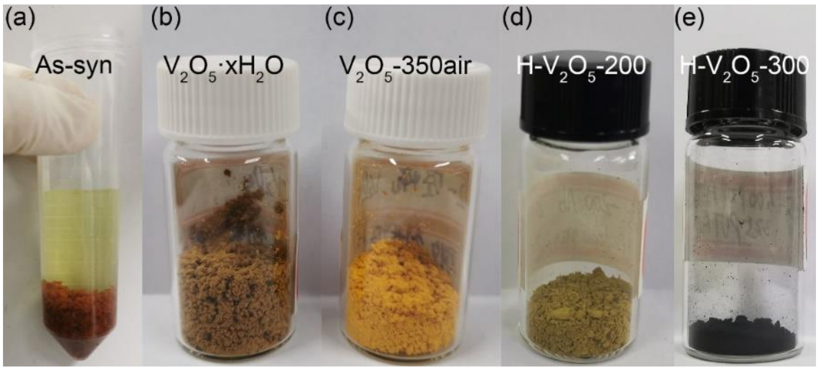

Fig. S1 Digital photos of **a** as-synthesized hydrothermal products, **b** $V_2O_5 \cdot xH_2O$, **c** V_2O_5-350air obtained by heat treatment of $V_2O_5 \cdot xH_2O$ at 350 °C in air, **d** H-V_2O_5-200 obtained by hydrogenation of V_2O_5-350air at 200 °C, and **e** H-V_2O_5-300 obtained by hydrogenation of V_2O_5-350air at 300 °C

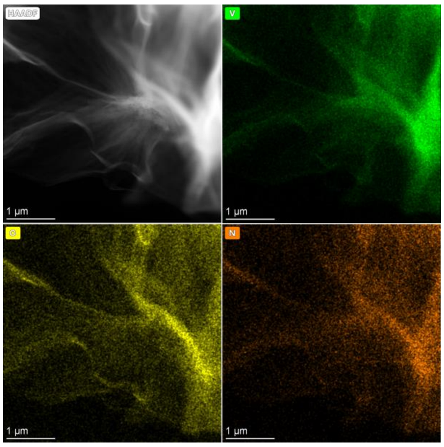

Fig. S2 HAADF image and the corresponding elemental mapping of pristine $V_2O_5 \cdot xH_2O$ nanosheets

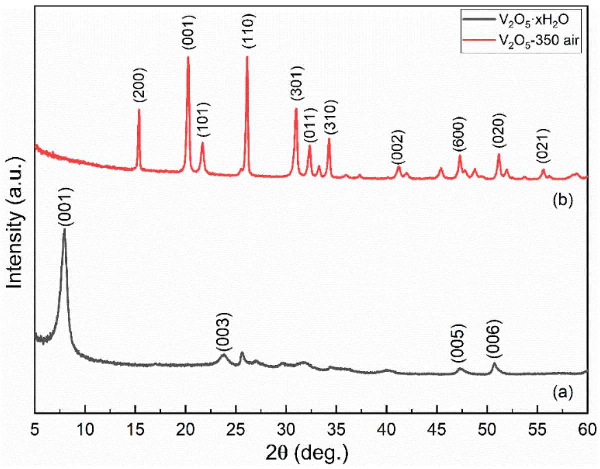

Fig. S3 XRD patterns of **a** $V_2O_5 \cdot xH_2O$ and **b** V_2O_5-350air

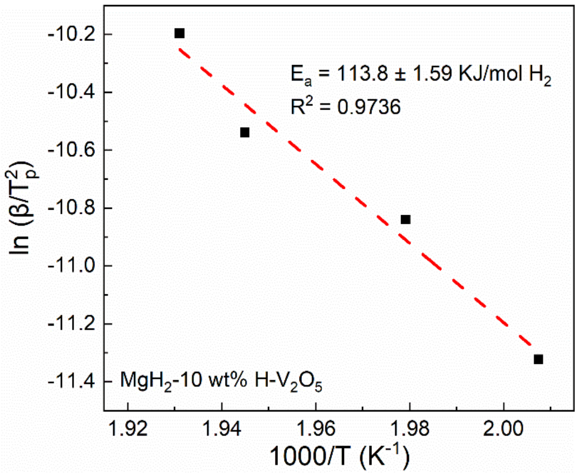

Fig. S4 Kissinger's plots of MgH_2-10 wt% H-V_2O_5

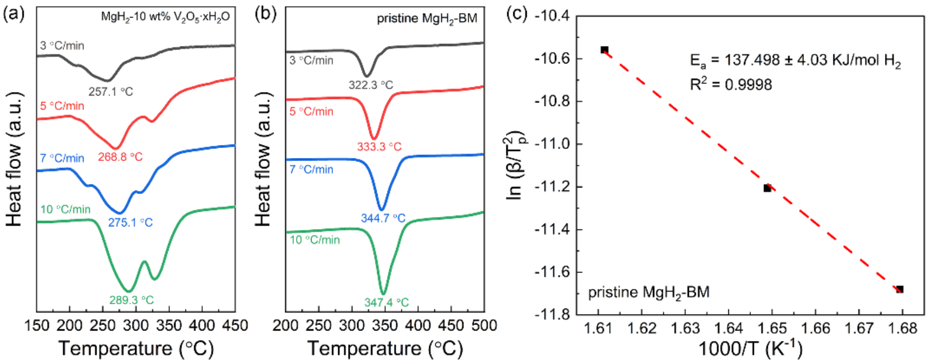

Fig. S5 The DSC curves of **a** MgH_2-10 wt% $V_2O_5·xH_2O$ and **b** pristine MgH_2-BM. **c** Kissinger's plots of pristine MgH_2-BM

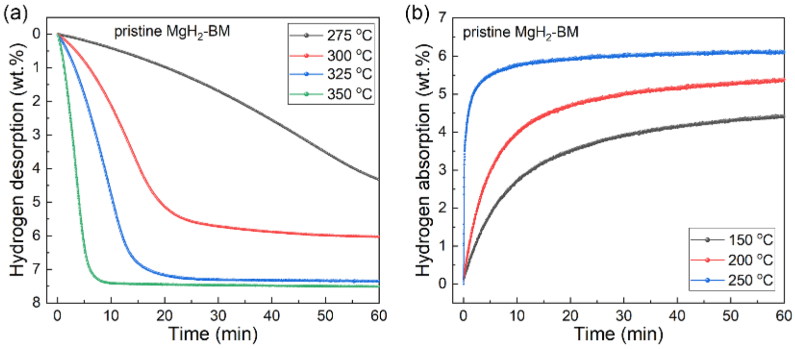

Fig. S6 The isothermal **a** desorption and **b** absorption curves of pristine MgH_2-BM

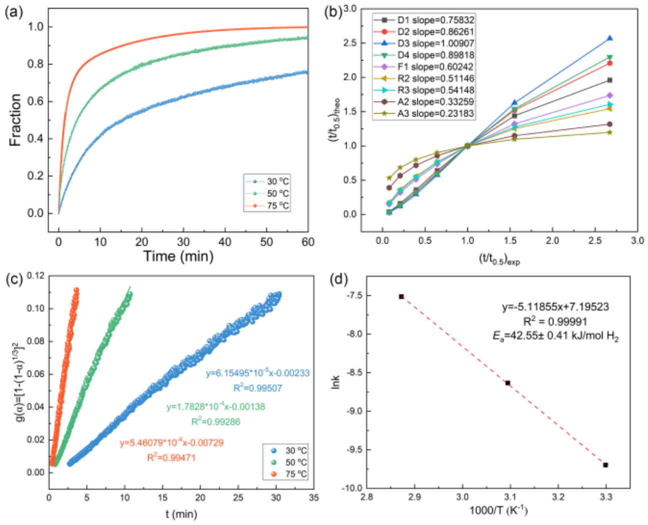

Fig. S7 a The extent of reaction curves of MgH_2-10 wt% H-V_2O_5 composites at 30, 50 and 75 °C. **b** $(t/t_{0.5})_{theo}$ vs. $(t/t_{0.5})_{exp}$ of composites at 50 °C for various kinetic models. **c** Time dependence of kinetic modeling equations g(α) for composites with 0.2 <α< 0.7 at different temperatures. **d** Calculation of the apparent activation energies according to the Arrhenius equation

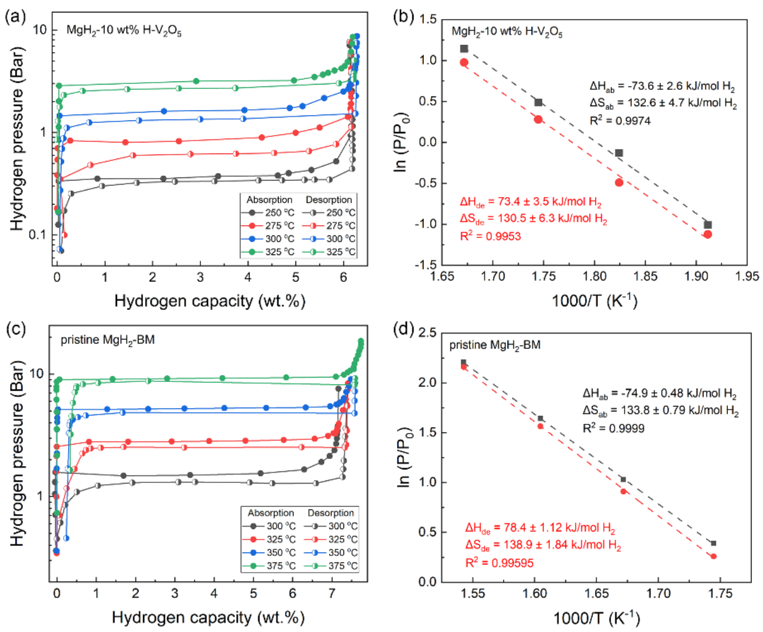

Fig. S8 PCI curves and van't Hoff plots of **a-b** MgH_2-10 wt% H-V_2O_5 and **c-d** pristine MgH_2-BM

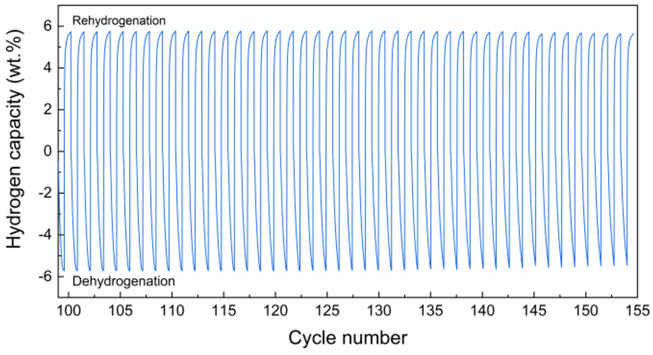

Fig. S9 De/re-hydrogenation cycle curves (100th-155th) of MgH_2-10 wt% H-V_2O_5

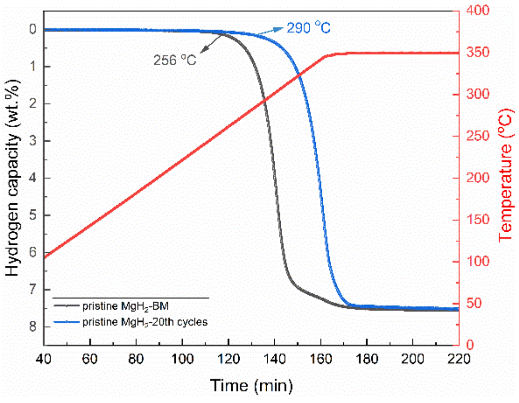

Fig. S10 TPD results of pristine MgH_2-BM upon cycling

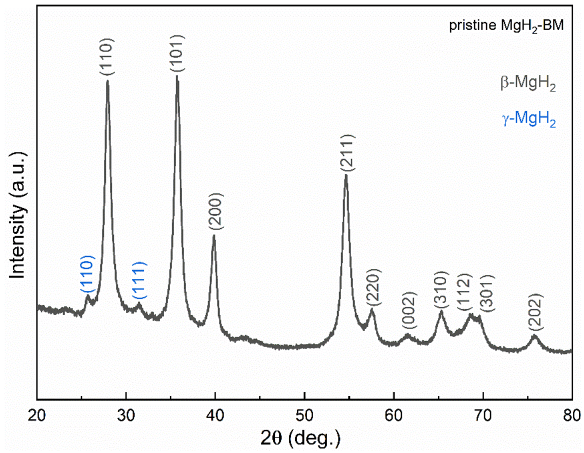

Fig. S11 XRD patterns of pristine MgH_2-BM

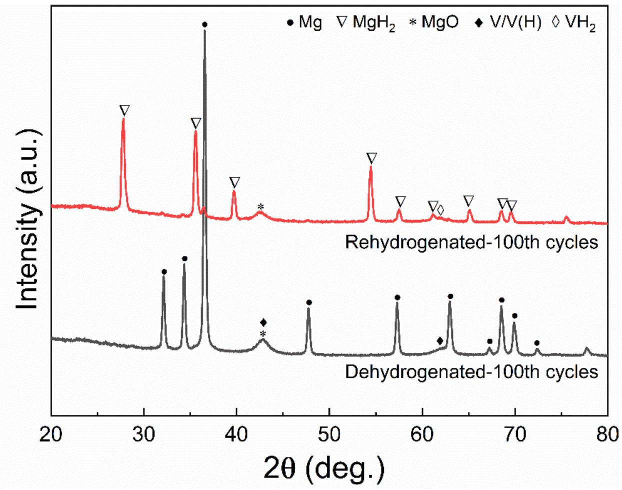

Fig. S12 XRD patterns of MgH_2-H-V_2O_5 after 100 cycles

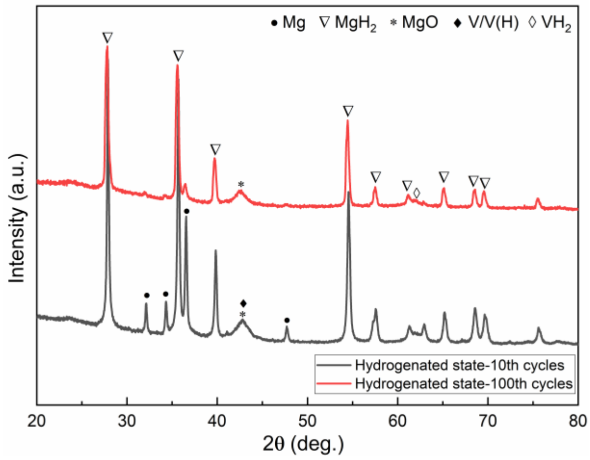

Fig. S13 XRD patterns of hydrogenated MgH_2-H-V_2O_5 after 10 and 100 cycles

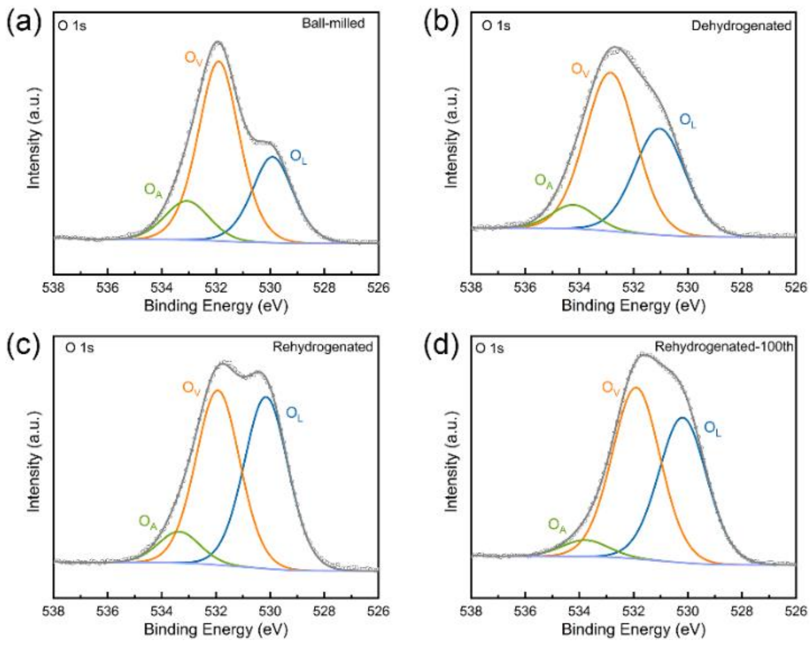

Fig. S14 High-resolution O 1s XPS spectra of MgH_2-10 wt% H-V_2O_5 at various states

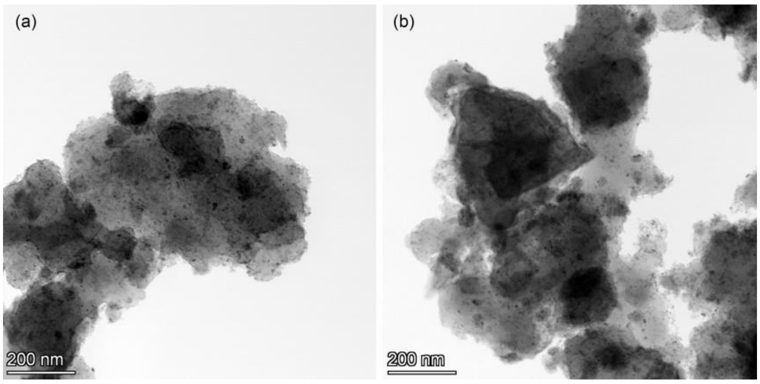

Fig. S15 Typical TEM images of the MgH_2-H-V_2O_5 composites after cycling tests

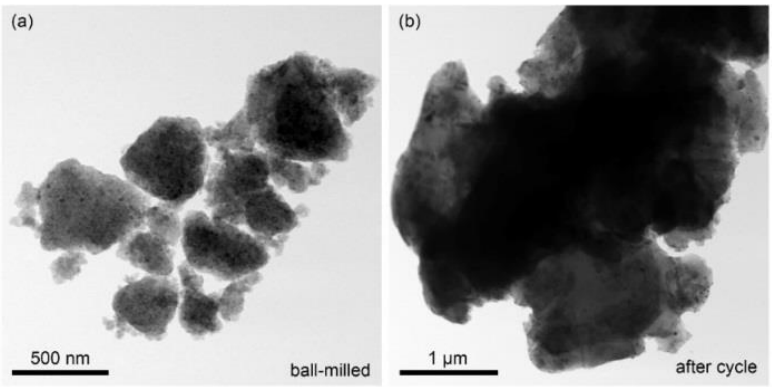

Fig. S16 Typical TEM images of pristine MgH_2-BM **a** before and **b** after ab/de-sorption cycles

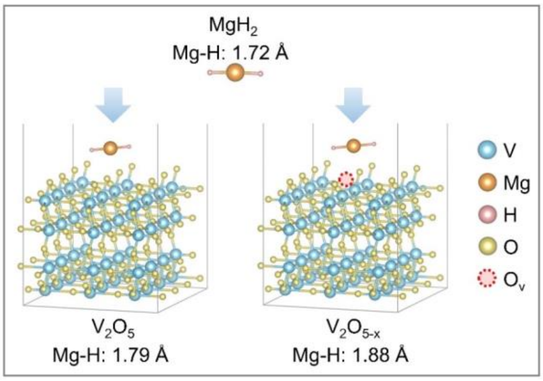

Fig. S17 Structure model of MgH_2 on V_2O_5 (001) plane/V_2O_{5-x} (001) plane and DFT calculation of the length of Mg-H bonds under the catalysis of V_2O_5 and V_2O_{5-x}, including MgH_2 for comparison

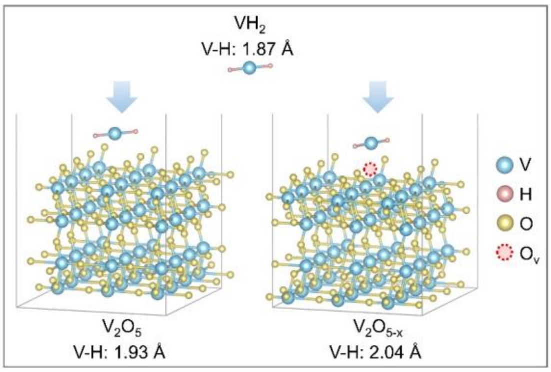

Fig. S18 Structure model of VH_2 on V_2O_5 (001) plane/V_2O_{5-x} (001) plane and DFT calculation of the length of V-H bonds under the catalysis of V_2O_5 and V_2O_{5-x}, including VH_2 for comparison

Table S1 The bond lengths and bond energy of Mg-H after MgH_2 adsorption at different active sites

Structures	Bond length (Å)	Bond energy (kJ mol^{-1})
MgH_2	1.72	92.39
MgH_2-V_2O_5	1.79	62.36
MgH_2-V_2O_{5-x}	1.88	40.93

Table S2 The bond lengths and bond energy of V-H after VH_2 adsorption at different active sites

Structures	Bond length (Å)	Bond energy (kJ mol^{-1})
VH_2	1.87	75.663
VH_2-V_2O_5	1.93	70.691
VH_2-V_2O_{5-x}	2.04	52.613
